# Magnetoliposomes Based on Shape Anisotropic Calcium/Magnesium Ferrite Nanoparticles as Nanocarriers for Doxorubicin

**DOI:** 10.3390/pharmaceutics13081248

**Published:** 2021-08-12

**Authors:** Beatriz D. Cardoso, Ana Rita O. Rodrigues, Manuel Bañobre-López, Bernardo G. Almeida, Carlos O. Amorim, Vítor S. Amaral, Paulo J. G. Coutinho, Elisabete M. S. Castanheira

**Affiliations:** 1 Physics Centre of Minho and Porto Universities (CF-UM-UP), Campus de Gualtar, 4710-057 Braga, Portugal; beatrizdiascardoso94@gmail.com (B.D.C.); ritarodrigues@fisica.uminho.pt (A.R.O.R.); bernardo@fisica.uminho.pt (B.G.A.); pcoutinho@fisica.uminho.pt (P.J.G.C.); 2 CMEMS-UMinho, Universidade do Minho, DEI, 4800-058 Guimarães, Portugal; 3 Life Sciences Department, INL—International Iberian Nanotechnology Laboratory, 4715-330 Braga, Portugal; manuel.banobre@inl.int; 4 Physics Department and CICECO, University of Aveiro, 3810-193 Aveiro, Portugal; amorim5@ua.pt (C.O.A.); vamaral@ua.pt (V.S.A.)

**Keywords:** magnetoliposomes, magnetic nanoparticles, shape-anisotropy, mixed ferrites, doxorubicin, magnetic hyperthermia

## Abstract

Multifunctional lipid nanocarriers are a promising therapeutic approach for controlled drug release in cancer therapy. Combining the widely used liposome structure with magnetic nanoparticles in magnetoliposomes allies, the advantages of using liposomes include the possibility to magnetically guide, selectively accumulate, and magnetically control the release of drugs on target. The effectiveness of these nanosystems is intrinsically related to the individual characteristics of the two main components—lipid formulation and magnetic nanoparticles—and their physicochemical combination. Herein, shape-anisotropic calcium-substituted magnesium ferrite nanoparticles (Ca_0.25_Mg_0.75_Fe_2_O_4_) were prepared for the first time, improving the magnetic properties of spherical counterparts. The nanoparticles revealed a superparamagnetic behavior, high saturation magnetization (50.07 emu/g at 300 K), and a large heating capacity. Furthermore, a new method for the synthesis of solid magnetoliposomes (SMLs) was developed to enhance their magnetic response. The manufacturing technicalities were optimized with different lipid compositions (DPPC, DPPC/Ch, and DPPC/DSPE-PEG) originating nanosystems with optimal sizes for biomedical applications (around or below 150 nm) and low polydispersity index. The high encapsulation efficiency of doxorubicin in these magnetoliposomes was proven, as well as the ability of the drug-loaded nanosystems to interact with cell membrane models and release DOX by fusion. SMLs revealed to reduce doxorubicin interaction with human serum albumin, contributing to a prolonged bioavailability of the drug upon systemic administration. Finally, the drug release kinetic assays revealed a preferable DOX release at hyperthermia temperatures (42 °C) and acidic conditions (pH = 5.5), indicating them as promising controlled release nanocarriers by either internal (pH) and external (alternate magnetic field) stimuli in cancer therapy.

## 1. Introduction

Liposomes are one of the most well-studied and well-investigated nanocarriers for drug delivery. Among the most acclaimed advantages are the biocompatibility, the capacity to carry large payloads, and the stabilization and protection of compounds from early inactivation, degradation, and dilution in circulation [1,2,3]. Regarding cancer therapy, liposomes have been used as a passive targeting strategy due to the gaps within the range of 100 to 780 nm between endothelial cells of tumor capillaries and the lack of lymphatic drainage—which is a phenomenon known as the Enhanced Permeability and Retention (EPR) effect [4]. However, it is recognized that intravenously injected liposomes are opsonized by plasma components (opsonins) and uptaken by the mononuclear phagocyte system (MPS) [5,6]. The modifications that occur in the nanocarriers surface can significantly affect their circulation time [7]. Polyethylene glycol (PEG) is a highly hydrophilic polymer that forms a hydrophilic shield on the liposomes surface and has been used to obtain sterically stabilized liposomes. PEGylated liposomes (also known as stealth liposomes) contribute to a prolonged systemic circulation by protecting the active moiety from the immune system, changing its physicochemical properties (as the hydrodynamic size) and providing hydrophilicity to hydrophobic drugs without changing their mechanism of action. Yet, it is reported that the liposomes’ ability to interact and release drugs in the target is reduced [1], and passive targeting needs to be complemented with other strategies.

The inclusion of magnetic nanoparticles into the liposomes core (forming solid magnetoliposomes, SMLs) results in a multifunctional lipid nanocarrier that can potentially allow its magnetic guidance, controlled drug release, and magnetic hyperthermia in target sites. Magnetic hyperthermia, besides causing direct damages in the cells, will allow overcoming tumor hypoxia. Temperature increase induces greater perfusion within the tumor, leading to higher internalization of chemotherapeutic drugs. It also promotes higher oxygenation, making the tumor more sensitive to the action of radiotherapy [8].

Superparamagnetic iron oxide nanoparticles have been intensively investigated and used in Magnetic Fluid Hyperthermia (MFH) [9,10,11], as the magnetization disappears once the external magnetic field is removed, avoiding particle aggregation and hence the possible embolization of the capillary vessels. These nanoparticles also exhibit remarkable magnetic heating properties that can be finely tuned by adjusting the composition, size, and shape. Even though magnetite (Fe_3_O_4_) and maghemite (γ-Fe_2_O_3_) are the most studied magnetic nanoparticles for biomedical applications, ferrous ions occurring mostly in magnetite seem to demonstrate a cytotoxic effect toward biological organisms [12]. Aiming at the synthesis of biocompatible magnetic nanoparticles, spinel ferrites (MFe_2_O_4_) without transition metals have been exploited. The distribution of metal cations in spinel ferrite sites are based on their ionic radii, size of the interstitial site, stabilization technique, and synthesis reaction conditions, resulting in tunable and unique electric, optical, and magnetic properties [13]. Magnesium ferrite nanoparticles (MgFe_2_O_4_) have shown strong magnetic properties [14] and magnesium-doped iron oxide superparamagnetic nanoparticles (in particular, Mg_0.13_-γFe_2_O_3_) revealed a high intrinsic loss power (ILP) in the range of 14 nHm^2^/kg [15]. It is reported that doping MgFe_2_O_4_ ferrite nanoparticles with a small fraction of calcium leads to an increase in magnetization [16]. Furthermore, cell viability tests have shown that calcium–magnesium mixed ferrite nanoparticles practically lack toxicity [17].

Since the heating efficiency is dependent on the crystalline anisotropy of magnetic nanoparticles, different morphologies can improve nanoparticles’ magnetic hyperthermia properties by optimizing their shape anisotropy. Cubic-shaped iron oxide magnetic nanoparticles have already shown an improved saturation magnetization and Specific Absorption Rate (SAR) when compared to the spherical counterparts [18]. With all of this in perspective, cubic-shaped calcium-substituted magnesium ferrite nanoparticles (specifically Ca_0.25_Mg_0.75_Fe_2_O_4_) can be good candidates to be included on a magnetoliposomes core, enhancing the magnetic response of the previously prepared spherical Ca_0.25_Mg_0.75_Fe_2_O_4_-based magnetoliposomes [19].

Doxorubicin (DOX) is an anthracycline antibiotic produced and extracted by *Streptomyces peucetius*, which has considerable significance in clinics as an anticancer drug. Despite its reported antineoplastic activity, the conventional use of doxorubicin (as a free drug) is limited by the associated adverse effects, pharmacokinetics limitations, cardiomyopathy, and the associated drug resistance in long-term anticancer treatments [20]. Potential treatment approaches that consider a size increase of the systemically administrated drug (at least, 5–10 nanometers in diameter) [21] and/or the encapsulation of DOX in multifunctional nanosystems that aims synergistic therapeutic effects in antitumor activity have been studied [22,23].

The main aim of this work was to develop a magnetic nanosystem and a novel method to produce it, with increased magnetic content in the inner compartment of lipid vesicles by decreasing its inherent amount of water. Magnetic, hyperthermia, and structural characterization confirmed the synthesis of shape-anisotropic cubic superparamagnetic nanoparticles with improved saturation magnetization and heating abilities. Their incorporation, as a solid core, in thermosensitive lipid vesicles resulted in magnetoliposomes that aimed at the controlled release of DOX at tumor microenvironments. The interaction of the drug-loaded nanosystem with Human Serum Albumin (HSA) was investigated to determine the ability of magnetoliposomes to protect the cargo from opsonization. Finally, the potential of SMLs as a new therapeutic approach for local controlled release under temperature (42 °C and 37 °C) and pH trigger (pH 5.5 and 7.4) was assessed.

## 2. Materials and Methods

Ultrapure water Milli-Q grade (MilliporeSigma, St. Louis, MO, USA) and spectroscopic grade solvents were used in all the synthesis procedures.

### 2.1. Synthesis of Cubic Ca_0.25_Mg_0.75_Fe_2_O_4_ Nanoparticles 

Shape-anisotropic cubic superparamagnetic nanoparticles of calcium-substituted magnesium ferrite (with 25% calcium), Ca_0.25_Mg_0.75_Fe_2_O_4_, were synthesized by a co-precipitation method adapted from [24]. First, 12.6 g of octadecylamine was heated until its melting point was reached (52.9 °C) under continuous magnetic stirring. An aqueous solution containing 0.75 mM of magnesium acetate tetrahydrate, 0.25 mM of calcium acetate hydrate, 2 mM of iron (III) citrate tribasic monohydrate, and 3.1 mM of oleic acid was added to the pre-heated octadecylamine. The mixture was heated at 10 °C per minute until reaching 200 °C and then left for 90 min at this temperature. The resulting precipitated nanoparticles were washed with ethanol by several cycles of centrifugation. Finally, the nanoparticles were calcined at 600 °C for 30 min (to remove octadecylamine and oleic acid residues) and washed with 10% ethanol in water, resulting in hydrophilic nanoparticles.

### 2.2. Solid Magnetoliposomes Preparation

A new solid magnetoliposomes (SMLs) preparation method was developed to better improve the reproducibility and the magnetic response of the previously used SMLs preparation method [19,25]. For magnetoliposomes preparation, the lipids dipalmitoylphosphatidylcholine (DPPC) (from Sigma-Aldrich, St. Louis, MO, USA) and 1,2-distearoyl-*sn*-glycero-3-phosphoethanolamine-*N*-[methoxy(polyethylene glycol)-2000] (ammonium salt) (DSPE-PEG2000, from Avanti Polar Lipids, Birmingham, AL, USA) were used. Solid magnetoliposomes were prepared by encapsulation of the synthesized nanoparticles into reverse micelle structures. For the first lipid layer, a thin film of DPPC with a final concentration of 1 mM (above critical concentration) was prepared by solvent evaporation under an ultrapure nitrogen flow. Then, 3 mL of heptane 99% (from Sigma-Aldrich, water content ≤ 0.1%) were added to the thin film and ultrasonicated at a power of 190 W for a time interval of 15 min to form reverse micelles with uniform size. After that, 1 μM of the previously synthesized dried nanoparticles was added to the ultrasonicated lipid formulation and subjected to 20 min of ultrasonication at 190 W to enforce the entrance of hydrophilic nanoparticles into the reverse micelles and form a magnetic cluster core. Magnetic decantation was used to purify the reverse micelles with a magnetic core. For that, a NdFeB N48 block magnet (Eclipse Magnetics Ltd., Sheffield, UK) with nickel-plated (Ni-Cu-Ni) coating was applied to the sample for one hour. After that time, the non-magnetic supernatant was discarded, and the remaining solvent was evaporated under an ultrapure nitrogen flow. The magnetic pellet was resuspended in ultrapure water.

**Figure 1:**
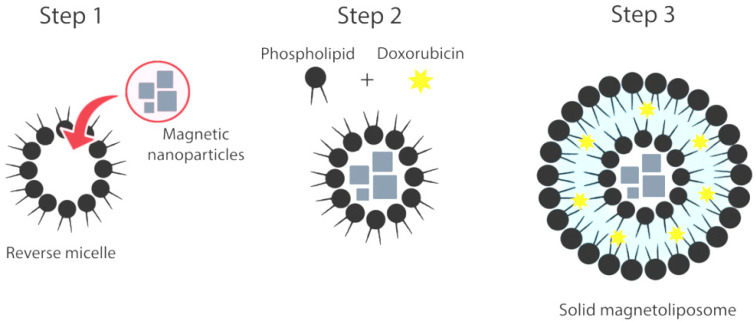
Schematic representation of the synthesis methodology of DOX-loaded solid magnetoliposomes.

Then, a total lipid concentration of 1 mM and was added to the pre-heated (>41 °C) reverse micelle solution to form the second lipid layer. Then, the resulting solid magnetoliposomes were washed and purified with ultrapure water by centrifugation. For magnetoliposomes containing cholesterol in the lipid bilayer, it was added, in this step, a lipid formulation that includes cholesterol (Ch) in a DPPC:Ch molar ratio of 7:3, respectively. To synthesize PEGylated magnetoliposomes, a lipid formulation of DPPC:DSPE-PEG in a molar ratio of 95:5 was employed. Doxorubicin was encapsulated in SMLs by co-injecting a DOX ethanolic solution (final concentration 2 × 10^−6^ M) with the addition of the second lipid layer under vortexing (Figure 1).

### 2.3. Preparation of Giant Unilamellar Vesicles (GUVs)

Giant Unilamellar Vesicles (GUVs) were used as models of cell membranes and were prepared using a procedure previously described [26]. First, by evaporation under a nitrogen flow, we prepared a film of 1 mM L-α-phosphatidylcholine (Soybean lecithin, from Sigma-Aldrich, St. Louis, MO, USA). After that, 20 µL of water was added to the film, and the resulted solution was kept at 45 °C for 45 min. Then, 3 mL of 0.1 M glucose solution was added, and the mixture was incubated at 37 °C for 2 h. Finally, GUVs solution was centrifuged at 14,000× *g* for 30 min at 20 °C to remove lipid aggregates and multilamellar vesicles (pellet).

### 2.4. Magnetic Measurements and Structural Characterization

Magnetic properties of Ca_0.25_Mg_0.75_Fe_2_O_4_ nanoparticles were investigated at room temperature in a Superconducting Quantum Interference Device (SQUID) magnetometer Quantum Design MPMS5XL (Quantum Design Inc., San Diego, CA, USA), using applied magnetic fields up to 5.5 T. The magnetization hysteresis loop measurements were made by fixing the temperature and measuring the magnetization at a series of different applied magnetic fields. 

The crystallographic state of the synthesized nanoparticles was investigated by X-ray diffraction (XRD) analyses using a conventional Philips PW 1710 diffractometer (Royal Philips, Amsterdam, The Netherlands), operating with Cu K_α_ radiation, in a Bragg–Brentano configuration. 

HR-TEM images of magnetic nanoparticles were recorded using a Transmission Electron Microscope JEOL JEM 2010F (JEOL Ltd., Tokyo, Japan) operating at 200 kV coupled to an Electron-Dispersive Spectroscopic analyzer (EDS). The processing of TEM images was performed using ImageJ software (version 1.52 p, National Institutes of Health (NIH), Bethesda, MD, USA).

The average hydrodynamic diameter and size distributions of the liposomal formulations were measured after preparation using Dynamic Light Scattering (DLS) equipment NANO ZS Malvern Zetasizer (Malvern Panalytical Ltd., Malvern, UK), using a He-Ne laser of λ = 633 nm, a detector angle of 173°, and a controlled temperature of 25 °C. The SMLs solutions (at a lipid concentration of 1 mM) were prepared in filtered ultrapure water and measured in polystyrene cells, with a solvent refractive index of 1.33, a material refractive index of 1.4, and an absorption coefficient of 0.001 L/m. Five independent measurements were taken for each sample, and the data were processed using Malvern Zetasizer software (Version 7.13).

The colloidal stability of SMLs was also assessed by DLS measurements. For that, 3 mL of DPPC-based SMLs aqueous solutions (at a lipid concentration of 1 mM) were stored at 4 °C for 30 days. Changes on hydrodynamic size and polydispersity index (PDI) of the samples were monitored during that time at the same conditions mentioned above.

### 2.5. Magnetic Hyperthermia Measurements 

The heating efficiencies of Ca_0.25_Mg_0.75_Fe_2_O_4_ nanoparticles were evaluated in a hyperthermia equipment DM 100 system, nB nanoscale Biomagnetics (Zaragoza, Spain) under an oscillating magnetic field at two different combinations of field strengths and frequencies: (a) H = 250 Gauss and f = 869 kHz and (b) H = 200 Gauss and f = 688 kHz, for a time range of one hour. An optical fiber sensor was placed in the mid-point of a glass container, which contained an aqueous solution of 1 mg of nanoparticles dispersed in 1 mL of water for temperature monitoring during the experiment. The container, in its turn, was placed in the mid-point of a copper coil. The temperature increase was logged as a function of time. Since the magnetic hyperthermia is strongly dependent on nanoparticles power absorption from the applied magnetic field, the specific absorption rate (SAR) can be calculated per unit mass of the nanoparticles used and is given the Equation (1),

(1) $$\mathrm{SAR}=C\times \frac{\Delta T}{\Delta t}\times \frac{m_{s}}{m_{m}}$$

where C is the specific heat capacity of suspension, $\frac{\Delta T}{\Delta t}$ is the initial slope of the curve, and $m_{s}$ and $m_{m}$ express the mass of suspension and magnetic material content in suspension, respectively. Even though the SAR is the most used way to characterize the heat capability generation, it does not allow comparing different experimental set-ups, because the SAR value strongly depends on the strength and frequency of the AC applied field. In order to normalize SAR values, the intrinsic loss power (ILP, nH m^2^/kg) has been suggested and is given by Equation (2),

(2) $$ILP=\frac{SAR}{H^{2}f}$$

where *H* is the field strength in kA/m, *f* is the frequency in kHz, and SAR should be introduced in W/kg [27].

### 2.6. Spectroscopic Measurements

Absorption spectra were measured with a double-beam Shimadzu UV-3600 Plus UV-Vis-NIR (Shimadzu Corporation, Kyoto, Japan) spectrophotometer using a 1 cm optical path length. Doxorubicin hydrochloride was used to prepare solutions in several solvents (which included water, methanol, acetonitrile, ethanol, ethyl acetate, and chloroform) at 1 × 10^−5^ M, and the spectra were recorded in the 300–600 nm wavelength range. Fluorescence spectra were acquired by a Fluorolog 3 (HORIBA Jobin Yvon IBH Ltd., Glasgow, UK) spectrofluorimeter, equipped with Glan–Thompson polarizers and double monochromators in excitation and emission. This equipment has a temperature-controlled cuvette holder (with continuous stirring), being adjusted to 25 °C, 42 °C, or 55 °C for the different performed experiments. Fluorescence spectra were corrected for the instrumental response of the system. The fluorescence quantum yields (Φ_F_) for doxorubicin in several solvents are the average of three independent measurements. 

Förster resonance energy transfer (*FRET*) occurs when a donor fluorophore in the excited state transfers energy to an acceptor moiety in the ground state in a non-radiative process. *FRET* efficiency is defined as the proportion of donor molecules that have transferred their excess energy to acceptor molecules. Experimentally, as previously reported, it can be calculated by taking the ratio of the donor integrated fluorescence intensities in the presence of an acceptor (*F_DA_*) and the absence of an acceptor (*F_D_*), following Equation (3) [28],

(3) $$\Phi_{FRET}=1-\frac{F_{DA}}{F_{D}}.$$

In turn, the values of FRET efficiency allow calculating the distance between the donor molecules and the acceptor ones (Equation (4)) [28],

(4) $$r_{AD}=R_{0}\;{\left[\frac{\;1-{\;\Phi }_{FRET}\;}{\Phi_{FRET}}\right]}^{1/6}$$

where *R*_0_ is the Förster radius (critical distance), which can be obtained by the spectral overlap, *J*(*λ*), between the donor emission and the acceptor absorption, according to the following relations (Equations (5) and (6)), with $R_{0}$ in Å, *λ* in nm, $\varepsilon_{A}\left(\lambda \right)$ in M^−1^ cm^−1^):

(5) $$R_{0}=0.2108\;{\left[k^{2\;}\Phi_{D\;}^{0}n^{-4}\;J\left(\lambda \right)\right]}^{1/6}$$

(6) $$J\;\left(\lambda \right)=\int_{0}^{\infty }I_{D}\left(\lambda \right)\varepsilon_{A}\left(\lambda \right)\lambda^{4}d\lambda$$

where *k*^2^ = 2/3 is the orientational factor assuming random orientation of the dyes, $\Phi_{D\;}^{0}$ is the fluorescence quantum yield of the donor in the absence of energy transfer, *n* is the refraction index of the medium, $I_{D}\left(\lambda \right)$ is the fluorescence spectrum of the donor normalized so that $\int_{0}^{\infty }I_{D}\left(\lambda \right)d\lambda =1$, and $\varepsilon_{A}\left(\lambda \right)$ is the molar absorption coefficient of the acceptor.

*FRET* assays were performed to confirm the formation of the lipid bilayer in the new method of SMLs preparation. For that, the first lipid layer included the energy acceptor rhodamine B labeled lipid, *N*-(lissamine rhodamine B sulfonyl)-1,2-dioleoyl-*sn*-3-phosphatidylethanolamine (Rh-DOPE) (ammonium salt) (from Avanti Polar Lipids, Birmingham, AL, USA)), and the second lipid layer included the energy donor nitrobenzoxazole labeled lipid NBD-C_12_-HPC (also from Avanti Polar Lipids, Birmingham, AL, USA). 

The steady-state fluorescence anisotropy, *r*, was calculated using Equation (7) to study DOX incorporation in SMLs. Several assays were performed for the different lipid formulations and temperatures.

(7) $$r=\left(\frac{I_{VV}-GI_{VH}}{I_{VV}+2GI_{VH}}\right)$$

Here, $I_{VV}$ and $I_{VH}$ are the intensities of the emission spectra obtained with vertical and horizontal polarization, respectively (for vertically polarized excitation light), and $G=I_{HV}/I_{HH}$ is the instrument correction factor, where $I_{HV}$ and $I_{HH}$ are the emission intensities obtained with vertical and horizontal polarization (for horizontally polarized excitation light).

### 2.7. Drug Encapsulation Efficiency and Drug-Loading Capacity

Doxorubicin encapsulation efficiency (EE%) in magnetoliposomes was estimated by absorbance measurements. After magnetoliposomes loading with doxorubicin, the aqueous solution of magnetoliposomes was magnetically decanted. The supernatant containing the non-encapsulated drug was used to determine the drug concentration by monitoring the absorbance at 480 nm (characteristic of DOX). Three independent measurements were performed for each system, and standard deviations (SD) were calculated. The drug encapsulation efficiencies of different lipid nanosystems were determined using Equation (8),

(8) $$EE\;\left(\%\right)=\frac{Initial\;drug\;concentration-drug\;concentration\;in\;the\;supernatant}{Initial\;drug\;concentration}\times 100$$

### 2.8. Interaction with Human Serum Albumin

The binding to human serum albumin (HSA) was studied by the titration of a fixed concentration of 0.2 mM of HSA aqueous solution (equivalent to HSA blood plasma concentration) with a freshly prepared aqueous solution containing the ligand (free doxorubicin, DPPC liposomes loaded with doxorubicin, and DPPC/DSPE-PEG liposomes loaded with doxorubicin). The experiment was carried out with liposomes instead of magnetoliposomes, since the presence of magnetic nanoparticles has a quenching effect on HSA fluorescence emission and thus strongly influences the results. In each increment (1 μL of ligand solution), the sample was left stabilizing at room temperature for 30 min. Each assay consisted of selectively measuring the fluorescent emission of tryptophan residues (excitation wavelength at 280 nm). The calculations were based on the changes of maximum fluorescence emission intensity found at 344 nm. The results were expressed by plotting fluorescence quenching of HSA emission as a function of ligands concentration. The emission spectra were recorded in the range of 290–700 nm, with an integration time of 1 s and the width of the slits set to 2 nm. The relative efficiency of HSA quenching can be described by Equation (9) [29],

(9) $$\%\;quenching=\frac{y_{max}{}^{n}}{1+\frac{k_{d}}{\left[ligand\right]}}$$

where $y_{max}$ is the maximum fluorescence quenching registered, *n* is the number of binding sites, and $k_{d}$ is the dissociation constant. The affinity between the protein and the ligand ($k_{b})\;$is inversely proportional to $k_{d}$ and can be expressed by 1/$k_{d}$.

### 2.9. Drug Release Kinetics and Mathematical Modeling of Release Profile

DOX release from SMLs was assessed in phosphate buffer at pH = 5.5 and 7.4 to simulate the drug release profile in the acidic tumor extracellular microenvironment and physiological fluids, respectively. The release studies were carried out at 37 °C and 42 °C (the latter to represent hyperthermia treatments). All measurements were done in triplicate. For this purpose, a reusable 96-well Micro Equilibrium Dialysis Device HTD 96b from HTDialysis, LLC (Gales Ferry, CT, US) was used with regenerated cellulose dialysis membranes. The DOX release was followed during 30 h. The samples were collected from the acceptor compartments at different time points and measured by fluorescence spectroscopy (λ_exc_ = 480 nm, in the range of 550–650 nm). The experimental doxorubicin release profiles were fitted to different kinetics models (Weibull, first-order and Korsmeyer–Peppas—see Supplementary Material) using Prism 8 software (GraphPad Software, La Jolla, CA, USA). 

## 3. Results and Discussion

### 3.1. X-ray Diffraction (XRD) Analysis

**Figure 2:**
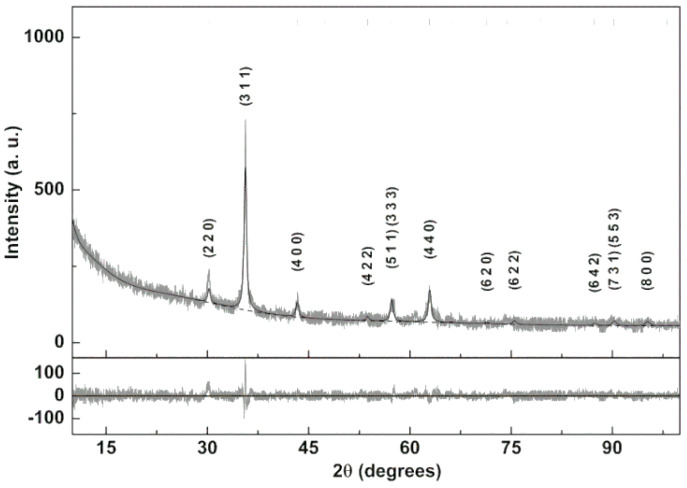
XRD pattern of the Ca_0.25_Mg_0.75_Fe_2_O_4_ nanoparticles and corresponding Rietveld analysis.

A new preparation method for superparamagnetic shape anisotropic Ca_0.25_Mg_0.75_Fe_2_O_4_ ferrite nanoparticles was developed. The corresponding XRD diffractogram is shown in Figure 2 and was analyzed using Profex software [30], which is based on BGMN [31] Rietveld calculations. The Ca/Mg mixed ferrite was defined through adaptation of magnesioferrite CIF file nr. 1011245 (space group Fd-3m:1), considering the distribution of cations across the tetrahedral and octahedral sites according to stoichiometry. The inversion degree was fixed to 0.825, as obtained previously for magnesioferrite [31]. A reasonable fit was obtained, with χ^2^ = 2.06 and R_P_ = 10.3. The obtained lattice parameter was 8.350 Å, which is near the value reported in the magnesioferrite CIF file (8.360 Å). The influence of preferred orientation on the relative intensity of the diffraction peaks was modeled by taking into account the symmetry of the ferrite space group through spherical harmonics, as is implemented in BGMN [31]. Still, the fit is not perfect, indicating the influence of the anisotropic structure of the prepared nanoparticles. A medium size of 20.9 ± 0.5 nm is estimated using the implementation of size broadening effects on BGMN [31].

### 3.2. Transmission Electron Microscopy

**Figure 3:**
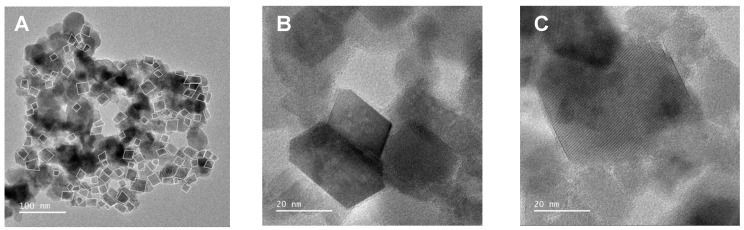

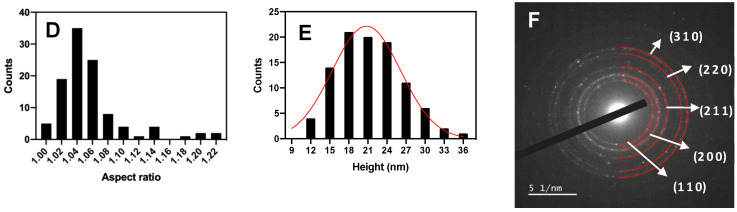
(**A**–**C**): High-Resolution Transmission Electron Microscopy (HR-TEM) images of Ca_0.25_Mg_0.75_Fe_2_O_4_ ferrite nanoparticles at different magnifications; (**D**): Aspect ratio distribution histogram; (**E**): Size histogram and distribution fit (R^2^ = 0.96) and (**F**): Small Area Electron Diffraction (SAED) image with indexed diffraction planes.

Transmission Electron Microscopy (TEM) analysis and small area electron diffraction (SAED) were carried out to determine the nanoparticles’ morphology, crystal structure, and their size distribution (Figure 3). From the statistical analysis of 106 outlined nanoparticles in Figure 3A, it was possible to extrapolate the mean length of major and minor axes of the particles and calculate the mean corresponding aspect ratio (height/width). In Figure 3B,C, it is possible to well distinguish elongated nanoparticles, which belong to a population with an aspect ratio slightly higher than 1. The aspect ratio distribution histogram is displayed in Figure 3D and reveals that nanoparticles generally present a cubic, slightly rectangular shape, resulting in a population with a mean aspect ratio of 1.04 ± 0.01. In addition, the size histogram was estimated considering the nanoparticles’ height (nm) and fitted to a Gaussian distribution, resulting in a population with a mean size of 20.8 ± 5.3 nm (Figure 3E), which is in accordance with the size estimated by XRD. The SAED ring pattern (Figure 3F) was indexed to a BCC structure, indexing (1 1 0), (2 0 0), (2 1 1), (2 2 0), and (3 1 0) from inside to outside.

### 3.3. UV-Visible Absorption

**Figure 4:**
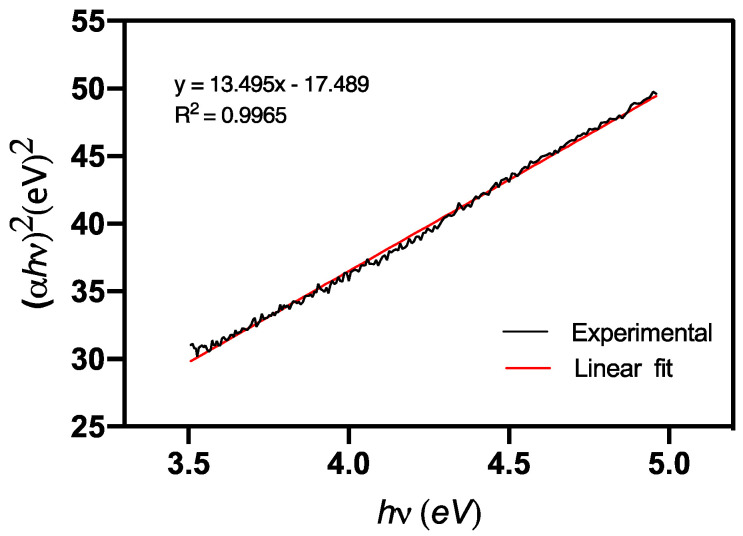
Tauc plot for shape-anisotropic Ca_0.25_Mg_0.75_Fe_2_O_4_ ferrite nanoparticles (the red line corresponds to the linear fit).

The optical properties of Ca_0.25_Mg_0.75_Fe_2_O_4_ ferrite nanoparticles were determined using the Tauc model for energy band gap estimation. The UV-visible absorption (or transmission) spectrum of the synthesized nanoparticles allows obtaining its optical band gap using a Tauc plot, which is given by Equation (10),

(10) $${\left(\alpha hv\right)}^{n}∼\left(hv-E_{g}\right)$$

where α is the absorption coefficient (proportional to the absorbance), n is an exponent that depends on the nature of the transition (being n = 2 for a direct semiconductor and n = 1/2 for an indirect semiconductor), and $E_{g}$ is the optical band gap. A linear relation was only obtained for n = 2, indicating that shape anisotropic Ca_0.25_Mg_0.75_Fe_2_O_4_ nanoparticles behave as an indirect semiconductor. The nanoparticles’ band gap was estimated from the intercept of ${\left(\alpha hv\right)}^{2}$ linear extrapolation with the Y-axis (Figure 4), resulting in the value of 1.29 eV. This band gap value is slightly lower than the previously obtained for spherical Ca_0.25_Mg_0.75_Fe_2_O_4_ ferrite (1.51 eV) [19], which is a result of structural changes. In the same previous work, there was a demonstrated increase of band gap values with the increase of calcium ratio in mixed ferrite nanoparticles [19].

### 3.4. Magnetic Properties 

Considering that magnetic hyperthermia is one of the aims of using solid magnetoliposomes in cancer therapy, it is fundamental to ensure that the magnetic nanoparticles (which constitute the SMLs core) present ideal features for biological application effectiveness.

In a previous work [19], the magnetic properties of spherical calcium-substituted magnesium ferrite nanoparticles (Ca_x_Mg_1-x_Fe_2_O_4_; *x* = 0.25, 0.50, 0.75) were studied, and a nonlinear effect on maximum magnetization, as a result of calcium substitution, was noticed. Although Ca^2+^ is a non-magnetic ion, its concentration highly influences the distribution of Fe^3+^ ions (which have a magnetic moment of 5 µB) between A (tetrahedral) and B (octahedral) sites. When calcium proportion in a ferrites structure assumes values of *x* > 0.05, Ca^2+^ migrates to B sites, and the distribution of calcium-substituted magnesium ferrite takes the form $\left(Ca_{x-y}^{2+}Fe_{1+x-y}^{3+}\right)\left[Mg_{1-x}^{2+}Ca_{y}^{2+}Fe_{1+x-y}^{3+}\right]O_{4}^{2-}$ (where the curve and square brackets indicate tetrahedral (A-sites) and octahedral (B-sites) sublattice, respectively), *y* and *x* representing the probability of migration of a small fraction of Mg^2+^ and Ca^2+^ ions to A-sites, respectively. The results revealed that the addition of a small proportion of calcium, and consequent substitution of magnesium in ferrite structure, leads to improved magnetic properties when compared to calcium ferrite and magnesium ferrite synthesized by the same preparation method. However, not only ion distribution but also size and shape anisotropy have the ability to influence nanoparticles’ overall magnetization. The shape of nanoparticles influences the anisotropy in magnetic nanomaterials, and the acicular shape is reported to have a favorable effect on the magnetic properties [32].

**Figure 5:**
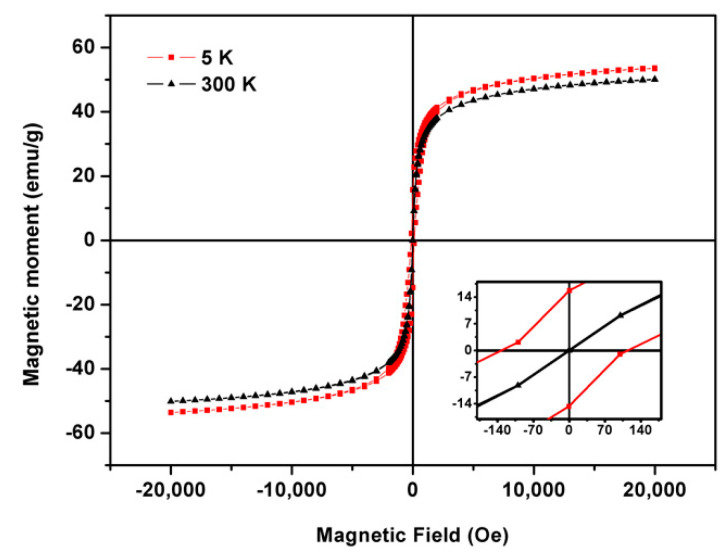
Magnetization hysteresis cycle of Ca_0.25_Mg_0.75_Fe_2_O_4_ ferrite nanoparticles at 5 K and 300 K. Inset: enlargement of the hysteresis loop in the low field region.

The magnetization measurements of shape-anisotropic Ca_0.25_Mg_0.75_Fe_2_O_4_ ferrite nanoparticles, upon calcination, were made at 5 K and 300 K. Figure 5 presents their magnetization hysteresis cycle, and the image inset shows a magnification of the low field region to make the coercivity more noticeable. The magnetic properties, including saturation magnetization (M_s_), coercive field (H_c_), and remnant magnetization (M_r_), are displayed in Table 1.

At room temperature, the hysteresis loop is almost closed, and the low values of coercivity and remnant magnetization are compatible with superparamagnetic behavior at high temperatures. The magnetic squareness value of the hysteresis cycle, which corresponds to the ratio between the remnant and saturation magnetization (M_r_/M_s_), allow to infer the presence of a superparamagnetic behavior. The magnetic squareness values here obtained are below 0.1 at 300 K, indicating that more than 90% of the magnetization was lost upon removing the magnetic field, corroborating the superparamagnetic behavior. The low values of M_r_/M_s_ (below 0.5) indicate a uniaxial anisotropy. Even though TEM images revealed nanoparticles with larger dimensions compared to the previously obtained, it is worth noticing that the superparamagnetic behavior is maintained. This happens since the critical grain size depends on the nanoparticles shape, and nanoarchitectures with shape anisotropy can remain in the single domain in larger dimensions than spherical nanoparticles [33].

As expected, the saturation magnetization values at T = 5 K are higher than those measured at T = 300 K due to the reduced thermal fluctuations at low temperatures. In addition, the coercivity value is higher for T = 5 K, due to the disappearance of the superparamagnetic behavior below the temperature at which the spins are blocked, that is, the blocking temperature. This noteworthy increase in coercivity was observed for all samples at 5 K and is attributed to the larger magnetic anisotropy at low temperatures. Here, the effect of thermal fluctuations of blocked moments across the anisotropy barrier is responsible for enhancing the nanoparticles’ coercivity values. Therefore, the absence of thermal fluctuations tends to make the magnetic moments isotropic for low temperatures, resulting in a coercivity increase [34].

The saturation magnetization of mixed ferrite nanoparticles strongly depends on the nanoparticles shape. Comparing the previously obtained spherical mixed ferrite nanoparticles of calcium and magnesium with the shape-anisotropic ones here synthesized (for the same proportion of calcium and magnesium ions) [19], a noticeable improvement in the magnetic properties was observed for the shape-anisotropic ones. Noh et al. [35] also compared the saturation magnetization between cubic and spherical Zn_0.4_Fe_2.6_O_4_ nanoparticles, the cubic ones reporting a higher saturation magnetization (M_s_ = 165 emu/g) than spherical particles (M_s_ = 145 emu/g), corresponding to 1.14 times improvement. In this work, a higher enhancement of 3.86 times in M_s_ was achieved, from a saturation magnetization of 12.98 emu/g (for the spherical nanoparticles [19]), to 50.07 emu/g (for shape-anisotropic nanoparticles with the same proportion of calcium and magnesium ions) (Figure 5). This enhancement comes from the effect of shape anisotropy on the ordering of surface atomic spins. Cubic nanoparticles have lower energy surface facets of the family (1 0 0), and the spherical presents different facets, resulting in a larger surface spin disorder broadly distributed in the sphere surface. In contrast, in the cubic, the disorder is dominant only at the corners. 

The obtained results point to a new and improved synthesis method for calcium-substituted magnesium ferrite nanoparticles, in which the given shape anisotropy does not compromise their superparamagnetic behavior. Their magnetic features also point to their suitability for SMLs magnetic guidance and effectiveness as magnetic mediators for magnetic hyperthermia in cancer therapy.

### 3.5. Magnetic Hyperthermia

**Figure 6:**
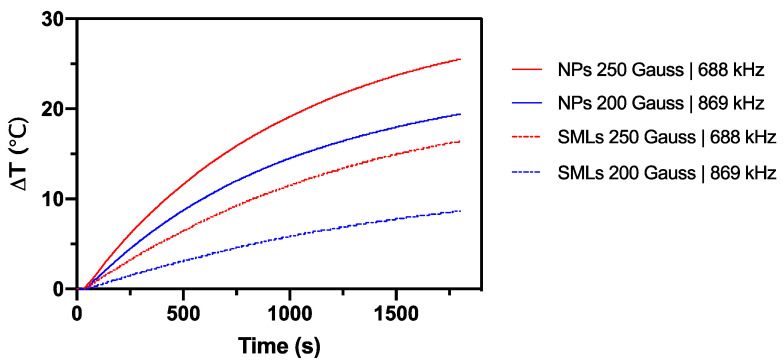
Magnetically-induced thermal response curve (temperature variation vs. time) of Ca_0.25_Mg_0.75_Fe_2_O_4_ nanoparticles (NPs) and SMLs for magnetic hyperthermia. One mg of nanoparticles were dispersed in 1 mL of water and subjected to an AMF with different field frequencies and strengths, over 30 min (1800 s).

To assess the hyperthermia potential of the synthesized cubic Ca_0.25_Mg_0.75_Fe_2_O_4_ ferrite nanoparticles and SMLs based on those nanoparticles, their heating efficiencies were evaluated under an oscillating magnetic field at two different combinations field strengths and frequencies: (a) H = 250 Gauss and f = 869 kHz and (b) H = 200 Gauss and f = 688 kHz. For that, 1 mg of Ca_0.25_Mg_0.75_Fe_2_O_4_ ferrite nanoparticles were dispersed in 1 mL of water. Figure 6 shows a time-dependent temperature of the aqueous suspension under the application of an alternate magnetic field. The synthesized nanoparticles demonstrated a heating profile compatible with mild hyperthermia. The nanoparticles revealed a large temperature variation over 30 min, with a ΔT = 25.5 °C and ΔT= 19.4 °C for H = 250 Gauss and f = 869 kHz and H = 200 Gauss and f = 688 kHz, respectively. The corresponding SMLs showed lower temperature variations, with a ΔT = 19.4 °C and ΔT = 16.48 °C for the same combinations of field strengths and frequencies. Hertg and Dutz have estimated that the product of the applied magnetic field and oscillating frequency, when exposed to small body regions, should be H·f ≤ 5 × 10^9^ A·m^−1^·s^−1^ [36]. Thus, the experiments here performed are slightly above this criterion limit.

As so, the nanoparticles must be further investigated to determine the conditions at which the reached temperatures are maintained at a mild hyperthermia threshold temperatures range, at the defined restriction limits for the safety and tolerance of patients. SAR (specific absorption rate) and ILP (intrinsic loss power) were estimated from the initial slope of the heating curve, following Equations (1) and (2), respectively. For nanoparticles, an SAR value of 34.66 W/g_Fe_ was estimated for an applied field of 200 G, whereas an SAR of 46.85 W/g_Fe_ was determined using an applied field of 250 G. The ILP estimated values were 15.69 nH∙m^2^/kg and 17.13 nH∙m^2^/kg, respectively. For SMLs, a SAR value of 12.26 W/g_Fe_ was estimated for an applied field of 200 G and 25.32 W/g_Fe_ for an applied field of 250 G; the calculated ILP values were 5.55 nH∙m^2^/kg and 9.25 nH∙m^2^/kg, respectively. This reduction of the heating performance of SMLs, relatively to the nanoparticles in the same conditions, can be explained by dipole interactions that are related to the nanoparticles cluster size. It was reported that at increased cluster sizes, the inherent nanoparticles shape anisotropy is gradually lost and, consequently, the heating capabilities are reduced [37]. Furthermore, Haase and Nowak [38] have demonstrated that an optimal particle concentration exists to achieve the highest heating power per sample.

### 3.6. Photophysical Properties of Doxorubicin in Solution

**Figure 7:**
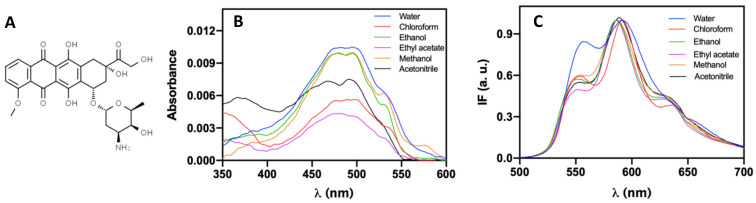
(**A**) Molecular structure of doxorubicin (C_27_H_29_NO_11_); (**B**) Absorption spectra of doxorubicin solutions, at 1 × 10^−5^ M, in several solvents; (**C**) Normalized fluorescence (at peak of maximum emission) spectra of doxorubicin (1 × 10^−6^ M, λ_exc_ = 480 nm) in several solvents.

Doxorubicin (DOX), as an anthracycline, is an amphiphilic molecule that possesses a fluorescent hydroxy-substituted anthraquinone chromophore and a hydrophilic aminoglycosidic side chain (Figure 7A) [39]. Considering that solute–solvent interactions have the ability to modify the intensity, position, and shape of absorption and emission bands, they can provide information about the structural changes of a molecule. The effect of several solvents on doxorubicin photophysical behavior was studied by its absorption and fluorescent profiles, with determination of maximum absorption wavelengths (λ_abs_), maximum emission wavelengths (λ_em_), molar absorption coefficients (ε), and fluorescence quantum yields (Φ_F_), all summarized in Table 2. The UV-vis. absorption spectra of DOX and normalized steady-state fluorescence in different solvents are shown in Figure 7B,C, respectively. 

Fiallo et al. [40] reported that the main band in the absorption spectra, around 480 nm, is associated with the π → π * transition polarized along the long (*y*) axis. In contrast, the band around 360 nm is associated with a partially forbidden *n* → π * transition (involving the three C=O groups). The maximum at 480 nm is given to the transition of a quinonoid structure [41]. These allowed transitions are responsible for the high molar absorption coefficients (in the range 10^3^–10^4^ M^−1^ cm^−1^) of doxorubicin in all the studied solvents.

Regarding the fluorescence spectra, two main peaks and a shoulder are distinguished around 560 nm, 590 nm, and 633 nm [42]. The doxorubicin fluorescence quantum yield demonstrated a higher value in water, Φ_F_ ≈ 20%, while in other solvents, it varies in the range of 4% (methanol, acetonitrile, and chloroform) to 6% (ethanol). These low fluorescence quantum yields can result from doxorubicin aggregation, with the formation of non-fluorescent dimers [43,44]. In addition, an evident change in spectral shape in water is observed, with loss of vibrational structure [45], the relative increase in first peak intensity, and band enlargement. This broadening suggests a weak charge-transfer character for the lowest electronic excitation in the ground-state geometry. A slight red shift is observed with increasing medium polarity, this bathochromic shift indicating a π → π * transition.

The intermolecular solute–solvent interactions have the capacity to induce changes in the profile of a spectrally active molecule [46]. The solvent contributions can be classified as (i) specific interactions, which include localized donor–acceptor interactions with specific orbitals and acid–base interactions involving hydrogen bonding; and (ii) non-specific interactions resulting from solvent acting as a dielectric continuum. Here, four physicochemical parameters were studied: the emission maxima (λ_em_), absorption maxima (λ_abs_), the Stokes’ shift (∆*ṽ*), and fluorescence quantum yield (Φ_F_) (see Supplementary Information). These results demonstrate that solvent dipolarity can be neglected for small variations on emission maxima, but solvent acidity, basicity, and polarizability play an active role in DOX emission maxima shift. In addition, solvent dipolarity is not a critical parameter to describe absorption maxima and Stokes’ shifts. Finally, fluorescence quantum yield values are not primarily influenced by the solvents’ basicity and dipolarity. Detailed analysis and information are presented in the Supplementary Material. The knowledge of fluorescence emission properties of DOX will be used in the next sections to monitor drug location and release from the developed nanosystems.

### 3.7. Characterization of Magnetoliposomes

#### 3.7.1. Synthesis of Solid Magnetoliposomes

**Figure 8:**
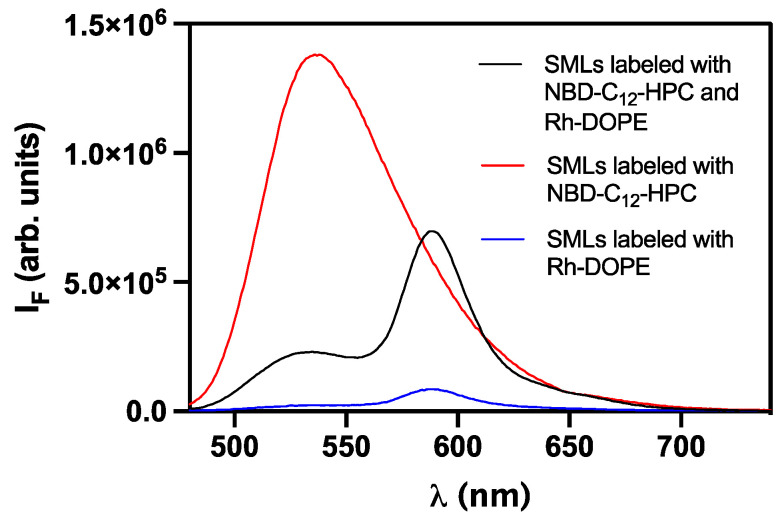
Fluorescence spectra (λ_exc_ = 470 nm) of SMLs with a DPPC bilayer labeled with only NBD-C_12_-HPC, with only Rh-DOPE and labeled with both NBD-C_12_-HPC and Rh-DOPE.

Herein, a new preparation method for solid magnetoliposomes was developed. This new approach begins with the preparation of DPPC reverse micelles in which, by ultrasonication, the previously synthesized magnetic nanoparticles are forced to enter into. A second DPPC layer is injected after magnetic decantation, resulting in a lipid bilayer vesicle with a magnetic core in a solid magnetoliposome architecture. Since the new methodology involves the independent addition of DPPC layers, the lipid bilayer formation was confirmed by Förster Resonance Energy Transfer (FRET), following the previously reported procedure [19]. The first lipid layer was labeled with Rhodamine B-DOPE (Rhodamine B as the energy acceptor), and the second lipid layer was labeled with NBD-C_12_-HPC (NBD as the energy donor). Once the second layer was added, the two fluorophores become close to each other, and the conditions for FRET occurrence are satisfied. Figure 8 shows the emission spectra of SMLs labeled with the energy donor NBD and the energy acceptor Rhodamine B, separately and together in a single system, which are all measured upon excitation of the donor (λ_exc_ = 470 nm). The pronounced decrease in the NBD emission, accompanied by an increase in Rhodamine B emission, evidences the energy transfer from excited NBD to Rhodamine.

The calculated FRET efficiency (Equation (3)) of 89.6% proves the probes’ proximity, as a strong energy transfer is observed from donor to acceptor. The lipid bilayer formation was corroborated by a donor–acceptor distance of 3.5 nm (from Equations (4) and (5)), since a lipid bilayer has a typical thickness between 7 and 9 nm [47]. FRET efficiency value is very similar to the previously obtained for magnetoliposomes containing spherical Ca_0.25_Mg_0.75_Fe_2_O_4_ nanoparticles [19], which reported an FRET efficiency of 87%. One of the main differences between the new synthesis method herein described and the one previously reported [19,25] is that the magnetoliposomes prepared by this new method do not present any amount of water in the inner compartment. As a result, the solid core makes the nanosystem less flexible (more rigid), which is translated into a decrease in lateral and rotational motion of lipids, improving the energy transfer between the probes.

#### 3.7.2. Dynamic Light Scattering and Transmission Electron Microscopy

**Figure 9:**
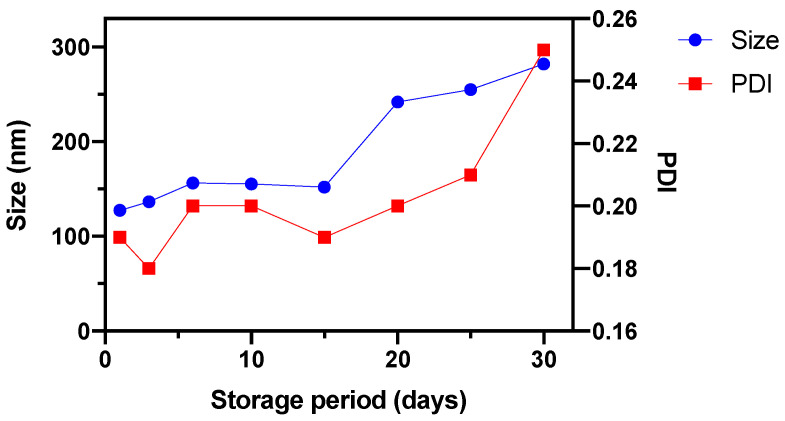
Hydrodynamic diameter and polydispersity (PDI) evolution of an aqueous solution of DPPC-based SMLs during storage at 4 °C for 30 days.

Considering the crucial role that the nanosystems’ inherent parameters (as size, polydispersity index, and shape) play on biological administration suitability and behavior, dynamic light scattering (DLS) and transmission electron microscopy (TEM) measurements were performed. DLS experiments allowed obtaining the size distribution of DPPC-based SMLs (Table 3) exhibiting an average hydrodynamic size of 117.5 ± 0.5 nm and a generally small polydispersity index (PDI). Correlation curves are presented in Figure S3 (Supplementary Material). The obtained average hydrodynamic size is slightly smaller than the previously obtained 127.3 ± 17 nm for SMLs containing spherical Ca_0.25_Mg_0.75_Fe_2_O_4_ nanoparticles [19], PDI values also being slightly lower. The stability of DPPC-based SMLs in storage at 4 °C was also monitored for 30 days. Figure 9 exhibits the size and PDI evolution of an aqueous solution of DPPC-based SMLs. This formulation is stable until day 15, with a small increase in size between days 15 and 20, to around 240 nm. A similar behavior was observed for PDI values, which increased to 0.25 on day 30 after storage. These results show that the nanosystems are completely stable for 15 days.

The encapsulation of low molecular weight molecules (as doxorubicin) in lipid vesicles increases the size of the administrated anticancer agent above the renal clearance threshold (≈40,000 Da), resulting in a reduction in kidney excretion and, consequently, blood half-life increase. It is reported that the encapsulation of doxorubicin in liposomes can improve its plasma half-life from 5–10 min (as a free drug) to 2–3 days (in liposomes) [48]. This increase in circulation times takes better advantage of the EPR effect, resulting in a more efficient and selective accumulation in tumor sites [49]. As mentioned before, the extravasation of liposomes to tumors is more effective at sizes below 200 nm [4]. DLS measurements confirm that SMLs hydrodynamic size is in conformity to the preferred size range. Considering that modifications in liposomal and micellar formulations have shown promising results in preventing the aggregation and opsonization of plasma proteins, PEGylated magnetoliposomes were also studied, exhibiting an average hydrodynamic diameter of 153.8 ± 0.8 nm and a low polydispersity index. The average size and PDI of PEGylated formulations were slightly higher than those of non-PEGylated ones (prepared by the same method). In fact, these results were not as expected, since we reported a general size reduction trend in PEGylated liposomal formulations. This size reduction is usually explained by an increased lateral repulsion caused by PEG molecules, which induces curvature in the lipid bilayer and consequent reduction in vesicle size [50]. The difference in size can be partially explained by a structural variation between liposomes and these magnetoliposomes, since the latter instead of an aqueous inner volume have a solid compartment constituted by a cluster of magnetic nanoparticles. The hydrodynamic diameter of SMLs with cholesterol (Ch) is similar to the values for PEGylated SMLs (151.4 ± 0.60 nm), however, with a lower PDI value (0.20 ± 0.01).

**Figure 10:**
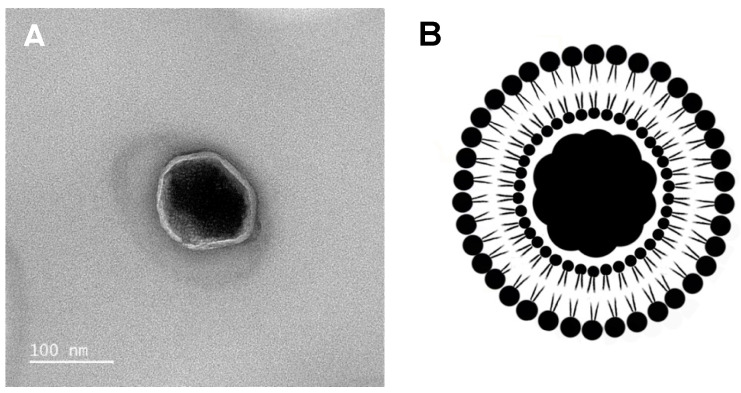
(**A**): HR-TEM image of a solid magnetoliposome based on Ca_0.25_Mg_0.75_Fe_2_O_4_ ferrite nanoparticles. (**B**): Schematic representation of a SML, characterized by the presence of a core (cluster of magnetic nanoparticles) covered with a lipid bilayer.

Transmission electron microscopy (TEM) allows characterizing the size and shape of SMLs. Figure 10A exhibits a single solid magnetoliposome, which is characterized by the presence of a nanoparticles cluster, highly contrasting with the bright thin DPPC lipid bilayer involving it. Using ImageJ software for image processing, several different segmented lines along the SML image were taken into account to measure the nanosystem mean diameter, resulting in mean diameter of 116.5 ± 5.5 nm. The same procedure was carried out to estimate the length of the lipid bilayer, resulting in a mean length of 8.3 ± 1 nm, complying with those reported in the literature and the obtained from FRET measurements. A schematic representation of solid magnetoliposomes was drawn from the clear TEM image and is presented in Figure 10B. An additional TEM image, at lower magnification (and different contrast), is presented in the Supplementary Material (Figure S2). The image reveals some dispersity in size, as inferred from DLS measurements (PDI near or above 0.2). It is also clear that the SMLs are not aggregated. The results prove the suitability of the new method here shown to originate magnetoliposomes with a typical structure, consisting of two DPPC monolayers surrounding an inner cluster core (with residual water content) of shape anisotropic nanoparticles.

#### 3.7.3. Doxorubicin Encapsulation in Solid Magnetoliposomes

**Figure 11:**
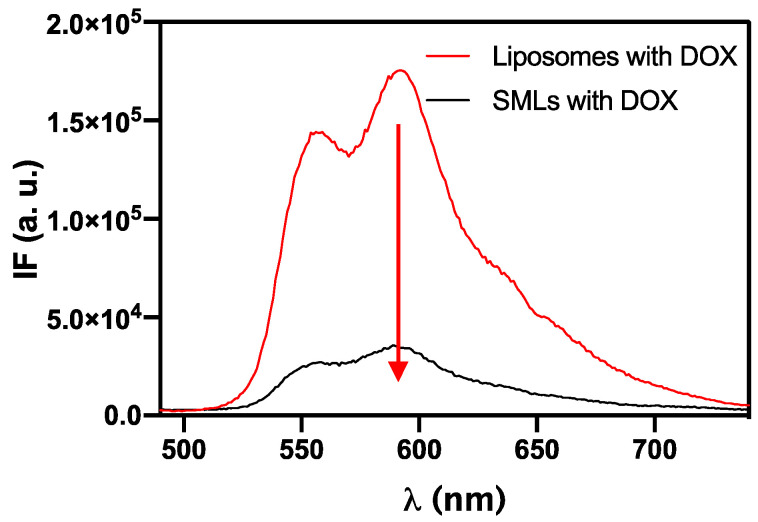
Fluorescence spectra (λ_exc_ = 480 nm) of doxorubicin (2 × 10^−6^ M) in liposomes (without magnetic nanoparticles) and in SMLs containing Ca_0.25_Mg_0.75_Fe_2_O_4_ magnetic nanoparticles.

The photophysical properties of doxorubicin were here exploited to study its encapsulation in SMLs and its location in the nanosystems. Figure 11 shows the emission spectra of doxorubicin, at the same concentration, in Ca_0.25_Mg_0.75_Fe_2_O_4_ based SMLs and in liposomes of the same lipid composition (without magnetic nanoparticles). 

The pronounced decrease in fluorescence emission of DOX in SMLs, comparatively to liposomes, is due to a quenching effect by the nanoparticles and confirms the incorporation of doxorubicin in the nanocarriers. This effect of fluorescence quenching promoted by the magnetic nanoparticles has been reported for several fluorescent antitumor drugs encapsulated in magnetoliposomes containing different types of magnetic nanoparticles [19,51,52,53,54]. The incorporation of doxorubicin in SMLs was further confirmed by fluorescence anisotropy measurements (Equation (7)) related to the rotational mobility of the fluorescent molecule. The results are shown in Table 4.

The fundamental fluorophore anisotropy (*r*_0_) corresponds to the maximum fluorescence anisotropy, and a value of 0.33 was reported for doxorubicin [42]. As expected, upon the encapsulation of DOX in SMLs (DPPC), room temperature anisotropy measurements registered values below *r*_0_ (*r* = 0.137), but typical of a fluorophore in a lipid bilayer, which confirms the DOX encapsulation in SMLs. Since the melting transition temperature of DPPC occurs at 41 °C, an increase in membrane fluidity is expected when the liquid-crystalline phase is attained. A lower fluorescence anisotropy value was observed at 55 °C, since the temperature increase implies an increased mobility of DOX aglycone in the bilayer, providing evidence of DPPC phase transition from the gel to the liquid-crystalline phase. 

Considering that the incorporation of cholesterol (present in most liposomal formulations used as drug carriers) and PEGylated lipids can significantly change the structural properties of a lipid bilayer, the anisotropy values of doxorubicin were measured for solid magnetoliposomes containing these modifiers. A considerable increase in anisotropy value, at 25 °C, in DPPC/DPSE-PEG SMLs was observed, indicating that DOX can be reliably retained in the membrane of PEGylated magnetoliposomes. At each condition, PEGylated SMLs showed higher anisotropy values than the other lipid formulations (Table 4), indicating a DOX location in a more exterior environment. In fact, the lipid bilayer fluidity varies with depth due to changes in membrane-free volume, showing a tendency to decrease from the liposome surface to the interior [55,56]. So, the higher *r* values may indicate that DOX is located more superficially, near PEG. Additionally, the significant decrease in anisotropy from 25 to 55 °C is an indicator that hyperthermia can enhance PEGylated SMLs’ ability to interact and release drugs in the target (due to the increase in membrane fluidity).

In the case of systems containing Ch, a general decrease in anisotropy was registered compared to non-modified vesicles. It is reported [57] that the addition of cholesterol in bilayers increases the apparent microviscosity of phosphatidylcholine (PC) membranes (up to 1.4 times) as a result of membrane-induced ordering of the PC acyl chains in the liquid phase. However, this effect is only verified to concentrations up to 20 mol% Ch. At higher content, its admixture decreases the microviscosity in the polar region of liposomes. Above 20 mol%, the region where cholesterol OH groups are located has a larger free volume available than in cholesterol-free membranes, resulting in a decreased fluorophore anisotropy [57]. This explains the smaller anisotropy values obtained for cholesterol-modified membranes.

Doxorubicin encapsulation efficiencies (EE%) were determined by the percentage of incorporated drug into SMLs relative to the initial amount of drug added (Equation (8)). Different lipid formulations with two different initial DOX concentrations were studied. As shown in Table 5, EE% decreased with increasing drug/lipid ratio. The EE% of non-modified DPPC-based SMLs varied between 72% ± 3% (for the initial concentration of 1 × 10^−4^ M) and 65% ± 8% (for the initial concentration of 2 × 10^−4^ M). At the same conditions, a slight decrease was evidenced from non-modified SMLs to PEGylated SMLs. These results suggest that PEGylation has a negligible effect on drug encapsulation efficiency. The lowest encapsulation efficiency was found in DPPC:Ch-based SMLs, which varied between 50% ± 2% and 48% ± 8%. The general high encapsulation efficiencies observed in all systems evidence the suitability of the new method herein presented for DOX encapsulation and the use of these nanocarriers for dual cancer therapy (by combining magnetic hyperthermia and chemotherapy).

**Figure 12:**
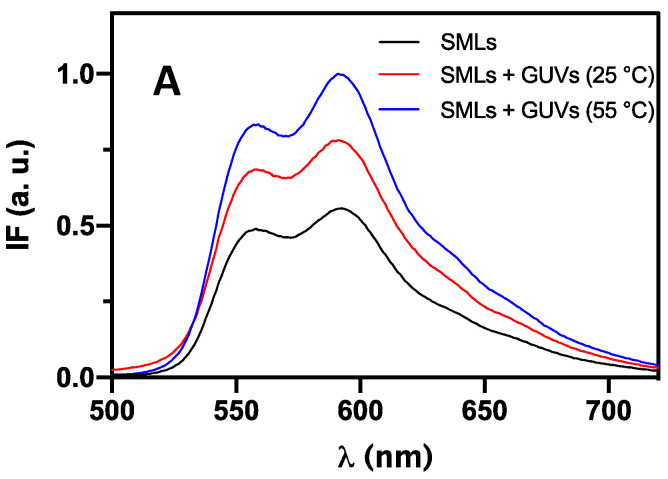

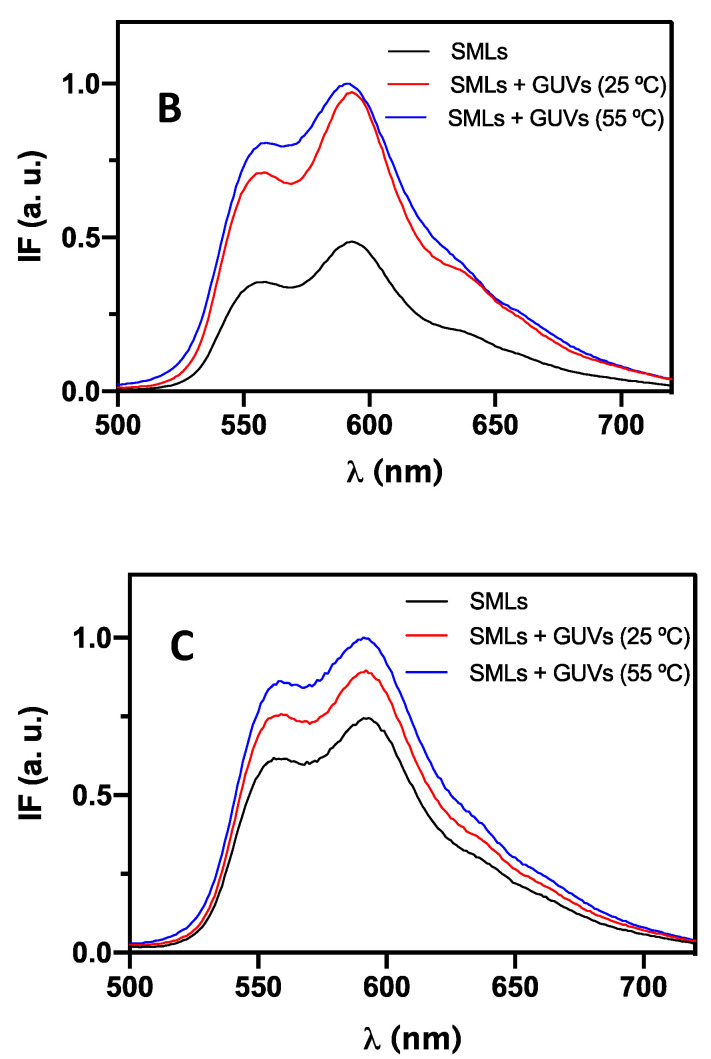
Fluorescence spectra (λ_exc_ = 480 nm) of doxorubicin in SMLs containing Ca_0.25_Mg_0.75_Fe_2_O_4_, before and after interaction with GUVs at 25 °C and 55 °C, for the different lipid formulations. (**A**) DPPC; (**B**) DPPC:DSPE-PEG; (**C**) DPPC:Ch.

Non-specific interaction between drug-loaded magnetoliposomes and GUVs (giant unilamellar vesicles, used as membrane models) was investigated to assess the ability of magnetoliposomes to release the content by fusion. Since doxorubicin fluorescence emission suffers a quenching effect from the magnetic nanoparticles that compose the SMLs core, this assay was performed by analyzing doxorubicin fluorescence variations before and after interaction with GUVs. Upon interaction, if fusion between the systems occurs, the DOX emission spectrum will reveal an unquenching effect due to the formation of a larger membrane and a corresponding increase in distance between the magnetic nanoparticles and DOX. The interest in DPPC-based SMLs is due to DPPC melting transition temperature, which is closely similar to the temperatures used in mild hyperthermia therapy. Therefore, the assays were also conducted at 55 °C to conclude the potential of magnetic hyperthermia to enhance fusion with cell membranes and the release capability of the solid magnetoliposomes. In all spectra of DPPC-based SMLs, DPPC:DSPE-PEG-based SMLs, and DPPC:Ch-based SMLs, an unquenching effect of DOX fluorescence is noticed upon SMLs’ interaction with GUVs (Figure 12). When the interaction takes place at 55 °C, the spectra revealed a more pronounced unquenching effect. These results prove that membrane fusion occurs and that the fusogenic capability is enhanced with an increase in temperature.

Doxorubicin anisotropy was also measured upon SMLs interaction with GUVs to corroborate the results (Table 4). It is important to notice that before interaction, DOX presented a lower anisotropy value in DPPC and DPPC:Ch lipid formulations than in DPPC:DSPE-PEG. Upon interaction with GUVs, DOX shows a slightly higher anisotropy in DPPC and DPPC:Ch-based SMLs than before interaction. In the case of DPPC:DSPE-PEG, a decrease in anisotropy was noticed. Since doxorubicin is located more superficially in PEGylated SMLs, anisotropy has an intermediate value between soy lecithin GUVs and DPPC:DSPE-PEG, proving interaction and fusion. These results can also explain the more prominent unquenching effect noticed in DPPC:DSPE-PEG spectra upon interaction with GUVs at 25 °C. In addition, above the DPPC phase transition temperature, an enhanced fusion between all systems occurs. The anisotropy values registered at 55 °C are rather low (around 0.07), which is the anisotropy value of doxorubicin in a saline PBS buffer [58]. These values point to a possible DOX release to the aqueous medium.

#### 3.7.4. Interaction with Human Serum Albumin 

The interaction between nanocarriers and Human Serum Albumin (HSA) has significance in investigating and designing new nanocarriers in vitro. HSA intrinsic fluorescence comes from the emission of tryptophan, tyrosine, and phenylalanine residues. However, HSA emission arises mainly from the Trp214 residue alone, which is located in the hydrophobic cavity. The interaction between HSA and lipid vesicles induces changes in protein conformation that result in a quenching effect of Trp fluorescence. Therefore, fluorescence quenching assays can give an insight into the interactions of doxorubicin and doxorubicin-loaded liposomes with plasma proteins that affect stability of the different lipid formulations under physiological conditions.

**Figure 13:**
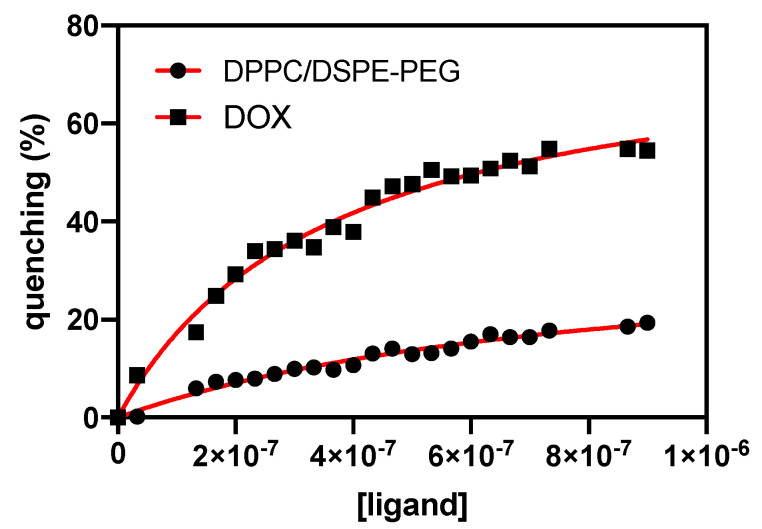
HSA fluorescence quenching (%) as a function of increasing doxorubicin concentration in free form and loaded in DPPC/DSPE-PEG-based liposomes. A nonlinear fit according to Equation (8) is presented (red lines).

The titration of an HSA aqueous solution (0.2 mM) with 1 μL of the different freshly prepared solutions (free doxorubicin, DOX-loaded DPPC liposomes, and DOX-loaded DPPC:DSPE-PEG liposomes) revealed a gradual HSA fluorescence drop, implying an interaction between the protein and doxorubicin or drug-loaded liposomes. The variation of HSA fluorescence quenching (%) with ligand concentration is shown in Figure 13, evidencing an apparent reduction of interaction between DOX and HSA when the drug is encapsulated in PEGylated nanocarriers. 

Fitting the results according to the nonlinear regression given by Equation (8), it was possible to calculate the dissociation constant (K_d_) and the number of specific binding sites and then estimate the binding constant (K_b_) (Table 6). The results revealed a large binding constant between DOX and HSA and 1.20 binding sites, indicating a significant binding of free doxorubicin to the blood plasma protein. A significant decrease in binding constants is observed upon the encapsulation of doxorubicin in DPPC-based and DPPC:DSPE-PEG-based liposomes. The obtained results suggest that DPPC and DPPC:DSPE-PEG nanosystems effectively protect DOX from interaction with plasma proteins. Even though the binding constant presents a higher value in PEGylated liposomes when compared to non-PEGylated ones, the number of available binding locations decreases. Thakur et al. [59] demonstrated that HSA penetrates DPPC liposomes, inducing an alteration in their packing order. This penetration is caused by hydrophobic interactions, which lead to the release of encapsulated payload. Thus, PEGylated vesicles can better protect the payloads, enhancing their bioavailability for longer time intervals, which is especially relevant for the effectiveness of EPR effect in tumors.

#### 3.7.5. Drug Release Kinetics and Mathematical Modeling of Release Profile 

**Figure 14:**
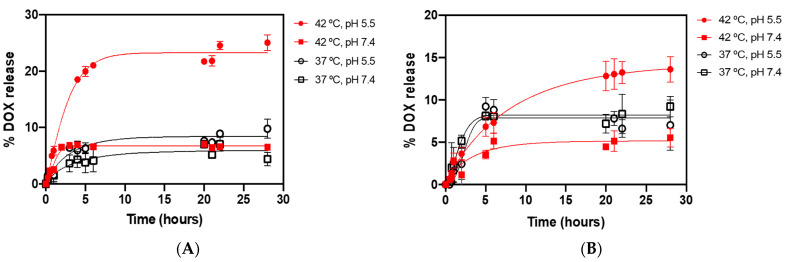
In vitro kinetic release profile of doxorubicin encapsulated in SMLs at different conditions of temperatures and pH, with the triplicate mean fitted to the Weibull kinetic model. (**A**) DPPC SMLs; (**B**) DPPC:DSPE-PEG SMLs.

Considering the complexity of cancer cells’ biology and the difficulties found along the treatments, it is essential to integrate combined and synergistic approaches to ensure a more effective, localized, and controlled therapy. The DOX release profiles from DPPC-based and DPPC:DSPE-PEG-based SMLs at different pH values (5.5 and 7.4) and temperatures (37 °C and 42 °C) are presented in Figure 14. For DPPC SMLs (Figure 14A), the results show that the drug release rate is highly dependent on pH and temperature. The release profile at 42 °C and pH = 5.5 stands out compared to the other combinations, exhibiting a burst release of 21.0 ± 0.4% in the initial six hours. The burst release is followed by a linear release phase, presenting a slow and controlled release profile that achieved the maximum release of 25 ± 2% at 28 h. Considering that 42 °C is above the DPPC transition temperature, there was an expected higher release rate at this temperature, considering the increase in permeability of magnetoliposomes membrane, which was verified at pH = 5.5. However, at pH = 7.4, the maximum release percentage is only 6.5 ± 0.2%, which is 3.5 times lower than the values obtained at pH = 5.5. These results provide evidence that acidic pH increases the hydrophilicity of DOX, contributing to an accelerated drug release, that is also verified at 37 °C. At physiological temperature, both pH conditions present slow-release kinetics, achieving a maximum release of 9 ± 1% at pH = 5.5 and 4 ± 1% at pH = 7.4. At physiological pH, DOX is protected by magnetoliposomes, and its release is delayed. In therapeutic conditions, the cardiotoxicity of the anthracyclines is dose-limiting, often leading to heart failure due to dilated cardiomyopathy years after exposure [60]. Willis et al. [61] demonstrated dose-dependent cardiac atrophy in mice at relatively low dose exposure. By delaying DOX release at physiological conditions, magnetoliposomes offer a protective effect over DOX-associated cardiomyopathy and other systemic adverse side effects. Liposomal doxorubicin was associated with a cardiac and gastrointestinal toxicity reduction, while maintaining a similar antitumor efficacy [62]. Recently, Swietch et al. [63] demonstrated that the medium pH has an impact on the affinity of DOX toward the carrier, the protonation of the anthracycline at acidic pH facilitating drug release. In addition, Chai et al. [64] observed an increased release rate of DOX at a decreased medium pH in DOX-loaded poly(lactic-co-glycolic acid) (PLGA) nanoparticles. The authors attributed the results to a strongly pH-dependent solubility, facilitating DOX release at low pH values.

A similar release kinetics behavior was found for DPPC:DSPE-PEG-based SMLs (Figure 14B), with a notable delayed release rate at 42 °C. PEGylated SMLs did not exhibit a burst release in the early hours, only achieving a maximum DOX release of 13 ± 1% at 28 h. These results indicate that the PEG matrix on SMLs’ surface acts as a barrier by reducing the diffusion of DOX into the surroundings. The increased sustained DOX release in PEGylated SMLs is indicative of a prolonged delivery period of the drug. 

Overall, the results point to a preferable DOX release at hyperthermia temperatures and acidic conditions, meaning that drug release may occur more readily in tumors. These are preliminary results of the behavior of the novel nanocarriers with a temperature and/or pH trigger, not considering the mechanical effect that superparamagnetic nanoparticles (subjected to an alternating magnetic field) would have on the active drug release. Considering that the purpose of controlled release systems is to maintain drugs in target sites at the desired concentration and control the drug release rate and duration, the sustained release ability of SMLs suggests a novel controlled release nanosystem. 

The doxorubicin release profile was fitted to three kinetic models, specifically Weibull, first-order, and Korsmeyer–Peppas (see Supplementary Material, Tables S3 and S4). The coefficients of determination (*R*^2^) indicate that Weibull is the best-fitting model to describe the overall release of DOX from both formulations. Since the Weibull model is empirical, it lacks kinetic basis information and its parameters do not present a physical meaning. Papadopoulou et al. [65] studied a link between the values of *b* and the diffusional mechanisms of the release, proposing that for *b* > 1, the drug transport follows a complex release mechanism; *b* ≤ 0.75 indicates Fickian diffusion (in either fractal or Euclidian spaces); 0.75 < *b* < 1 indicates a combined mechanism (Fickian diffusion and Case II transport). Table S3 (DPPC SMLs) shows that at 42 °C, both pH conditions present a *b* value higher than 1, evidencing a complex mechanism for DOX release. At 37 °C, it is verified that 0.75 < *b* < 1, evidencing a Fickian diffusion. Table S4 (DPPC:DSPE-PEG SMLs) indicates that *b* < 0.75 in all the conditions, pointing to a Fickian diffusion. The results are indicative of a change in the mechanism of DOX release when functionalizing DPPC SMLs with PEG. 

Similarly to the Weibull model, the first-order model revealed a higher *R*^2^ for the assays performed at 42 °C and pH = 5.5 in both formulations, suggesting that the amount of released DOX is proportional to the remaining drug in the nanocarrier. The Korsmeyer–Peppas model presents the worst coefficient of determination values. The *n* values are under the threshold value of 0.43, unless in the PEGylated SMLs at 42 °C and pH = 5.5. However, *n* < 0.5, indicating a diffusion-controlled release mechanism [66]. Since DOX is encapsulated in nearly spherical-shaped nanosystems, values of *n* ≤ 0.45 are representative of a Fickian diffusion mechanism [67]. The large variations of *R*^2^ found at 42 °C in DPPC SMLs (when compared to the ones obtained by the Weibull model) indicate a complex drug release mechanism instead of only a diffusion-controlled release.

**Figure 15:**
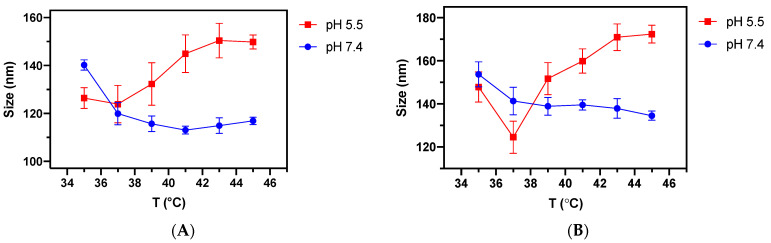
Size variation of DOX-loaded SMLs with increasing temperature at different pH values. (**A**) DPPC SMLs; (**B**) DPPC/DSPE-PEG SMLs. Mean size values and standard deviation are from triplicate assays.

The morphology evolution of DOX-loaded DPPC and DPPC/DSPE-PEG SMLs as a function of temperature at both pH values were studied by DLS measurements (Figure 15). Overall, it was noticed that the SMLs size tends to gradually decrease at pH = 7.4 with increasing temperature. At pH = 5.5, both formulations revealed an opposite variation in size, with a tendency to increase with rising temperature. These results show that SMLs become more unstable at acidic conditions and above the DPPC transition temperature, corroborating the DOX release profiles presented above.

## 4. Conclusions

In this work, cubic-shaped Ca_0.25_Mg_0.75_Fe_2_O_4_ nanoparticles, with an average size of approximately 20 nm, were successfully synthesized by a co-precipitation method, their saturation magnetization being considerably higher than that of the spherical counterparts. These nanoparticles were incorporated in solid magnetoliposomes prepared by a new method. The technique proved to produce SMLs with different lipid formulations, with sizes suitable for biomedical applications, encapsulating the chemotherapeutic drug doxorubicin. DOX-loaded SMLs revealed the ability to fuse with model membranes (GUVs), with enhanced fusogenic capabilities at higher temperatures. The interaction of DOX with HSA protein is strongly reduced when the drug is loaded in the liposomal nanocarriers, indicating a protective effect that improves drug bioavailability. DOX release profiles revealed an enhanced release under acidic conditions and mild hyperthermia temperature. A more sustained release was obtained for PEGylated SMLs, pointing to a prolonged delivery from PEGylated nanocarriers. 

Overall, the results indicate that the combination of shape-anisotropic magnetic nanoparticles and thermosensitive lipid bilayers is a promising approach for dual cancer therapy, by combining chemotherapy and magnetic hyperthermia. To the best of our knowledge, it is the first time that solid magnetoliposomes were prepared using cubic calcium-substituted magnesium ferrite nanoparticles.

Future work will focus on magnetically controlled drug release, with determination of the strength, frequency and AMF application time for optimal doxorubicin release. In addition, the synergistic effect between chemotherapy and magnetic hyperthermia will be investigated in human tumor cell lines. 

## 5. Patents

Portuguese Patent Nr. 115474, “Nanossistema magnético e método para produzir o nanossistema”, published in “Boletim de Propriedade Industrial” Nr. 2021/04/30, Portugal. International Patent PCT/IB2020/053947—“Magnetic Nanosystem and Method to Produce the Nanosystem” submitted.

## Figures and Tables

**Table 1:** Coercive field (H_C_), maximum magnetization (M_s_), remnant magnetization (M_r_), and ratio M_r_/M_s_ for cubic Ca_0.25_Mg_0.75_Fe_2_O_4_ ferrite nanoparticles.

	T (K)	H_C_ (Oe)	M_s_ (emu/g)	M_r_ (emu/g)	M_r_/M_s_
M (H)	300	19.93	50.07	1.41	0.03
	5	128.64	53.65	15.66	0.29

**Table 2:** Maximum absorption (λ_abs_) and emission wavelengths (λ_em_), molar absorption coefficients (ε) and fluorescence quantum yields (Φ_F_) for doxorubicin in several solvents.

Solvent	λ_abs_/nm (ε/10^4^ M^−1^ cm^−1^)	λ_em_/nm	Φ_F_^a^
Water	487 (0.50)	591	0.20 ± 0.02
Methanol	488 (0.49)	586	0.04 ± 0.01
Acetonitrile	480 (0.38)	588	0.04 ± 0.01
Ethanol	489 (0.49)	587	0.06 ± 0.01
Ethyl acetate	484 (0.21)	587	0.05 ± 0.01
Chloroform	488 (0.28)	592	0.04 ± 0.01

^a^ Relative to fluorescein in 0.1 M NaOH aqueous solution.

**Table 3:** Hydrodynamic diameter (D_H_) and polydispersity (PDI) at 25 °C. Results as mean ± SD.

SMLs Lipid Formulation	D_H_ (nm)	PDI
DPPC	117.5 ± 0.5	0.17 ± 0.01
DPPC/DSPE-PEG	153.8 ± 0.8	0.27 ± 0.01
DPPC/Ch	151.4 ± 0.6	0.20 ± 0.01

**Table 4:** Steady-state fluorescence anisotropy (*r*) values for doxorubicin in SMLs with different lipid formulations, in SMLs upon interaction with GUVs and in GUVs (for comparison). Total lipid concentration: 1 × 10^−3^ M; DOX: 2 × 10^−6^ M.

Nanosystem	Lipid Formulation	Temperature ( °C)	*r*
SMLs	DPPC	25	0.137
	DPPC:DSPE-PEG (95:5)		0.206
	DPPC:Ch (7:3)		0.075
	DPPC	55	0.100
	DPPC:DSPE-PEG (95:5)		0.133
	DPPC:Ch (7:3)		0.043
GUVs	Soybean lecithin	25	0.116
		55	0.058
SMLs + GUVs	DPPC	25	0.166
	DPPC:DSPE-PEG (95:5)		0.187
	DPPC:Ch (7:3)		0.139
	DPPC	55	0.081
	DPPC:DSPE-PEG (95:5)		0.121
	DPPC:Ch (7:3)		0.098

**Table 5:** Encapsulation efficiency, EE (%), and corresponding encapsulated DOX concentration, in solid magnetoliposomes containing Ca_0.25_Mg_0.75_Fe_2_O_4_ at different concentrations of DOX. Standard deviation is from triplicate drug encapsulation assays.

	Initial DOX Concentration	
Lipid Formulation	1 × 10^−4^ M	2 × 10^−4^ M
DPPC	72% ± 3% (7.23 × 10^−5^ M)	65% ± 8% (1.31 × 10^−4^ M)
DPPC:DSPE-PEG (95:5)	70% ± 22% (7.04 × 10^−5^ M)	52% ± 19% (1.05 × 10^−4^ M)
DPPC:Ch (7:3)	50% ± 2% (5.05 × 10^−5^ M)	48% ± 8% (9.75 × 10^−5^ M)

**Table 6:** Dissociation constant ($k_{d}$), binding constant ($k_{b}=1/k_{d}$), and number of binding locations (*n*) of DOX, DPPC liposomes, and DPPC:DSPE-PEG liposomes to HSA.

	*k*_*d*_ (M)	*k*_*b*_ (M^−1^)	*n*	*R* ^2^
Free DOX	3.69 × 10^−9^	27.1 × 10^7^	1.20	0.993
DPPC	2.54 × 10^−6^	3.93 × 10^5^	2.27	0.977
DPPC:DSPE-PEG	14.47 × 10^−7^	1.18 × 10^6^	1.78	0.978

## Data Availability

Not applicable.

## Supplementary Materials

The following are available online at https://www.mdpi.com/article/10.3390/pharmaceutics13081248/s1, 1. Photophysical properties of doxorubicin in solution: Table S1: Estimated coefficients (y_0_, a_α_, b_β_, c_π *_), their standard errors and correlation coefficients (R) for the multiple linear regression analysis of λ_em_, λ_abs_, ∆ṽ and Φ_F_ as a function of the Kamlet–Taft solvent scale. The regression coefficients are expressed in nm for λ_em_ and λ_abs_, in cm^−1^ for ∆ṽ and in 10^−2^ for Φ_F_; Table S2: Estimated coefficients (*y*_0_, *a_SA_*, *b_SB_*, *c_SP_*, *d_SdP_*), their standard errors and correlation coefficients (*R*) for the multiple linear regression analysis of λ_em_, λ_abs_, ∆*ṽ* and Φ_F_ as a function of the Catalán solvent scales. The regression coefficients are expressed in nm for λ_em_ and λ_abs_, in cm^−1^ for ∆*ṽ* and in 10^−2^ for Φ_F_; Figure S1: Linear relationship of the experimental and calculated (**A**) λ_em_ (*R* = 0.99); (**B**) λ_abs_ (*R* = 0.98); (**C**) ∆*ṽ* (*R* = 0.97); and (**D**) Φ_F_ (*R* = 0.96) of Doxorubicin obtained by the multiple linear regression analysis according to Catalán solvent scale. 2. TEM image and DLS correlation curves of SMLs: Figure S2: TEM image of solid magnetoliposomes at a lower magnification. Figure S3: DLS correlation curves for solid magnetoliposomes. (**A**): SMLs of DPPC; (**B**): SMLs of DPPC/Ch; (**C**): SMLs of DPPC/DSPE-PEG. 3. Drug release kinetics and mathematical modeling of release profile: Table S3: Obtained constant values by the fitting of each mathematical model to the kinetic data and respective coefficient of determination (*R*^2^), according to the temperature and pH variation for DPPC-based SMLs; Table S4: Obtained constant values by the fitting of each mathematical model to the kinetic data and respective coefficient of determination (*R*^2^), according to the temperature and pH variation for DPPC/DSPE-PEG-based SMLs.
