# Supplementary material for: Magnetoliposomes Based on Shape Anisotropic Calcium/Magnesium Ferrite Nanoparticles as Nanocarriers for Doxorubicin
Source: Pharmaceutics. 2021 Aug 12;13(8):1248. doi: 10.3390/pharmaceutics13081248 (PMC8401618; doi:10.3390/pharmaceutics13081248)
Supplement: Supplementary file 1 [file pharmaceutics-13-01248-s001.zip › pharmaceutics-1314104-supplementary.pdf]

pharmaceutics

Supplementary Material: Magnetoliposomes Based on Shape Anisotropic Calcium/Magnesium Ferrite Nanoparticles as Nanocarriers for Doxorubicin

Beatriz D. Cardoso, Ana Rita O. Rodrigues, Manuel Bañobre-López, Bernardo G. Almeida, Carlos O. Amorim, Vítor S. Amaral, Paulo J. G. Coutinho and Elisabete M. S. Castanheira

1. Photophysical properties of doxorubicin in solution

The behavior of the drug doxorubicin is determined not only by its molecular structure, but also by the nature of solvent interactions established in biological systems. Considering that solvent highly influences the photophysical behavior of a molecule, the systematic analysis of its spectral features can give relevant physicochemical insight. Therefore, the understanding of microenvironment influence on spectroscopic properties of doxorubicin provides information that can be extrapolated to drug-solvent interactions in biological systems.

Solvatochromic methods have been widely used to study solvent effects on photophysical properties (e.g. absorption maxima, emission maxima and Stokes' shift) [1,2]. The multi-parameter Kamlet-Taft {α, β, π*} [3,4] and the Catalán {aSA, bSB, cSP, dSdP} [5] solvent scale, by solvatochromic data analysis, provide the contribution of interactions to the polarity effect on spectroscopic characteristics. Both models by taking in account non-specific and solute-solvent interaction, give a quantitative description of the solvatochromic shifts [6]. Catalán solvent scale (unlike the Kamlet-Taft solvent scale) has the advantage to separate non-specific solvent effects into two independent parameters (polarizability and dipolarity).

The Kamlet-Taft solvent scale uses the π*, α and β parameters (polarity/polarizability, acidity and basicity, respectively, of a given solvent) to study solute-solvent interactions, following Equation S.1. The basicity and acidity (characterized by hydrogen bond acceptor ability and hydrogen bond donor ability, respectively) are specific interactions, whereas non-specific interactions include polarity and polarizability, characterized by the solvent ability (by its dielectric effect) to stabilize a charge or a dipole.

$$y = y_0 + a_\alpha \alpha + b_\beta \beta + c_{\pi*} \pi * \quad (S.1)$$

The estimated coefficients y_0, a_α, b_β and $c_{\pi*}$ and the corresponding correlation coefficients for the multilinear regression analyses of the emission maxima, absorption maxima, Stokes' shift and fluorescence quantum yield, using the Kamlet-Taft solvent scale, are displayed in Table S1. The Kamlet-Taft π*, α and β solvatochromic parameters were taken from [7].

The influence of solvents, on the same parameters, was also studied using the linear solvation energy relationship model of Catalán [8], given by Equation S.2 and Catalán parameters were taken from [9,10].

$$y = y_0 + a_{SA}\,SA + b_{SB}\,SB + c_{SP}SP + d_{SdP}SdP \quad (S.2)$$

where y_0 is the physicochemical property in the gas phase, SA is the solvent acidity, SB is the solvent basicity, SP is the solvent polarizability and SdP is the solvent dipolarity. In this model, these solvent parameters are independent (but complementary) and responsible for multiple types of solute-solvent interactions. In this equation, a_{SA}, b_{SB}, c_{SP}

and d_{SdP} are the regression coefficients that describe the sensitivity of the respective parameter to the solute-solvent interaction mechanisms.

Both models take into account both specific and non-specific interactions, providing a quantitative description of the solvatochromic shifts [6]. However, Catalan's model allows the separation of non-specific solvent effects into polarity and polarizability. The results of the multiple regressions are displayed in Table S2. The multi-linear analysis of the obtained λ_{em} data of DOX as a function of {α, β, π*} shows a lower correlation coefficient (R = 0.87), when compared to the correlation obtained by the use of {SA, SB, SP, SdP} (R = 0.99). This difference is significant, since in Kamlet-Taft model the solvent (di)polarity and polarizability effects are combined in π* parameter and in Catalan's model they are split in SP and SdP parameters. Figure S1-A shows the linear relation between the λ_{em} calculated values using Catalán solvent scale *versus* the corresponding experimental values. Also, in Kamlet-Taft analysis, if {α, β} are used as independent variables (Equation S.3), the obtained correlation coefficient (R = 0.86) is similar to the original fit (R = 0.87).

$$y = y_0 + a_\alpha\, \alpha + b_\beta\, \beta \tag{S.3}$$

Table S1. Estimated coefficients (y_0, a_α, b_β, c_{π^*}), their standard errors and correlation coefficients (R) for the multiple linear regression analysis of λ_{em} , λ_{abs}, $\Delta\tilde{v}$ and Φ_F as a function of the Kamlet-Taft solvent scale. The regression coefficients are expressed in nm for λ_{em} and λ_{abs}, in cm^{-1} for $\Delta\tilde{v}$ and in 10^{-2} for Φ_F.

	y_0	a_α	b_β	c_{π^*}	R
λ_{em}	589.(5) ± 4	-7.(8) ± 5	1.(1) ± 3	3.(3) ± 6.64	0.87
λ_{em}	591.(7) ± 1	2.(6) ± 1	-9.(6) ± 3		0.86
λ_{abs}	494.(5) ± 7	-8.(6)± 8	11.(4) ± 6	-16.(2)± 11	0.81
$\Delta\tilde{v}$	[32.(4) ± 2]×10^{-5}	[1.(4) ± 2]×10^{-5}	[-4.(5) ± 1]×10^{-5}	[7.(9) ± 3]×10^{-5}	0.92
Φ_F	0.07 ± 0.02	0.05 ± 0.03	-0.0006 ± 0.0188	-0.0009 ± 0.0266	0.89

In Catalán analysis, the larger c_{SP} coefficient estimate value compared to the estimated {a_{SA}, b_{SB}, c_{SP}, d_{SdP}} in the analysis of λ_{em} (equation S.4) indicates that the change of λ_{em} maxima can be attributed to a change in polarizability of doxorubicin environment. In order to validate this, the relation between y = λ_{em} and the polarizability parameter SP was studied (equation S.5).

$$y = y_0 + a_{\mathrm{SA}}\, \mathrm{SA} + b_{\mathrm{SB}}\, \mathrm{SB} + d_{\mathrm{SdP}}\mathrm{SP} \tag{S.4}$$

$$y = y_0 + c_{\mathrm{SP}}\mathrm{SP} \tag{S.5}$$

The correlation coefficient of 0.99 confirms the linear relationship in Table S2. However, only neglecting solvent dipolarity (Equation S.4, with {SA, SB, SP} as independent variables) produces a fit (*R* = 0.98) with almost the same quality as the original fit (*R* = 0.99). These results demonstrate that solvent dipolarity can be neglected for small variations on λ_{em}, but solvent acidity, basicity and polarizability play an active role in the shift of DOX emission maxima.

The multi-linear analysis of λ_{abs} data as a function of {SA, SB, SP, SdP} presents a better correlation (*R* = 0.98) compared to the analysis as a function of {α, β, π*} (*R* = 0.81) (Table S2).

Table S2. Estimated coefficients (y_0, a_{SA}, b_{SB},, c_{SP}, d_{SdP}), their standard errors and correlation coefficients (R) for the multiple linear regression analysis of λ_{em} , λ_{abs}, $\Delta\tilde{\nu}$ and Φ_F as a function of the Catalán solvent scales. The regression coefficients are expressed in nm for λ_{em} and λ_{abs}, in cm^{-1} for $\Delta\tilde{\nu}$ and in 10^{-2} for Φ_F.

	y_0	a_{SA}	b_{SB}	c_{SP}	d_{SdP}	R
λ_{em}	565.(4) ± 19	1.(1) ± 1	-2.(2) ± 4	32.(7) ± 21	2 ± 5	0.99
λ_{em}	572.(4) ± 5	1.1 ± 0.7	- 3.(7) ± 1	25.(4) ± 7		0.98
λ_{em}	564.(9) ± 5			35.(3) ± 8		0.89
λ_{abs}	390.(4) ± 38	9 ± 1	24.78 ± 8.63	114.(7) ± 41	8.(4) ± 10	0.98
λ_{abs}	419.(4) ± 13	9.(5) ± 1	18.77 ± 3.92	84.(5) ± 17		0.97
λ_{abs}	489.(5) ± 7				-4.(3) ± 9	0.17
$\Delta\tilde{\nu}$	(69 ± 22)×10^{-5}	(-4 ± 1) ×10^{-5}	(-11 ± 5)×10^{-5}	(-39 ± 24)×10^{-5}	(-3 ± 6)×10^{-5}	0.97
$\Delta\tilde{\nu}$	(59.(6) ± 6)×10^{-5}	(-4 ± 8) ×10^{-5}	(-9 ± 1)×10^{-5}	(-29 ± 8)×10^{-5}		0.96
$\Delta\tilde{\nu}$	(34.(5) ± 3)×10^{-5}				(2 ± 4)×10^{-5}	0.17
Φ_F	0.3 ± 0.2	-0.01 ± 0.01	-0.02 ± 0.03	-0.28 ± 0.18	-0.08 ± 0.07	0.94
Φ_F	0.05 ± 0.02	-0.01 ± 0.01		0.02 ± 0.01	- 0.01 ± 0.03	0.88
Φ_F	0.11 ± 0.05	-0.02 ± 0.01		- 0.09 ± 0.07		0.80

A good linear relationship (R = 0.98) was found between the experimental λ_{abs} values and the λ_{abs} calculated values according to Equation S.2, using the estimated values of a_{SA}, b_{SB}, c_{SP} and d_{SdP} (Figure S1-B). Similar to what was verified for λ_{em}, the multi-linear fit of λ_{abs} data as a function of {SA, SB, SP} as independent variables, according to Equation S.4, revealed a correlation coefficient of 0.97, which is nearly the same as the original fit (Equation S.2). The same analysis was made as a function of {SdP}, according to Equation S.6, and a poor relationship was found between λ_{abs} and SdP (R = 0.17). The results confirm that solvent dipolarity is not critical, primarily, in λ_{abs} shifts.

$$y = y_0 + d_{\mathrm{SdP}}\mathrm{SdP} \quad \text{(S.6)}$$

For Stokes' shift ($\Delta\tilde{\nu}$), Kamlet-Taft analysis produces a good fit (R = 0.92), but Catalán analysis (R = 0.97) outperforms it. In Figure S1-C it is represented the linear relationship between experimental and calculated $\Delta\tilde{\nu}$ obtained by the multiple linear regression analysis according to Catalán model. In Catalán analysis, if {SA, SB, SP} are used as independent variables in the linear equation to fit y = $\Delta\tilde{\nu}$ (Equation S.4), the correlation coefficient (R = 0.96) is similar to the one obtained as a function of {SA, SB, SP, SdP}. This result indicates that solvent dipolarity is not a critical parameter to describe Stokes' shift. This is also corroborated by the poor correlation coefficient (R = 0.17) obtained by the linear fit of y = $\Delta\tilde{\nu}$ as a function of SdP (Equation S.6).

The multi-linear analysis of Φ_F data as a function of {α, β, π*} is less adequate to describe Φ_F values, since it presents a lower correlation (R = 0.89), when compared to the analysis as a function of {SA, SB, SP, SdP} (R = 0.94). The linear relationship between experimental and calculated values, according to Catalán solvent scale, is represented in Figure S1-D. The Catálan analysis of Φ_F as a function of {SA, SP, SdP} as independent variables (Equation S.7) gives rise to a relatively good fit (R = 0.88). This result is an indication that small changes on Φ_F are not primarily influenced by solvents basicity. Furthermore, the same analysis was made as a function of {SA, SP}, with an R value of 0.80. This result indicates that solvents dipolarity can be neglected for small Φ_F variations.

$$y = y_0 + a_{\mathrm{SA}}\,\mathrm{SA} + d_{\mathrm{SdP}}\mathrm{SP} + d_{\mathrm{SdP}}\mathrm{SdP} \quad \text{(S.7)}$$

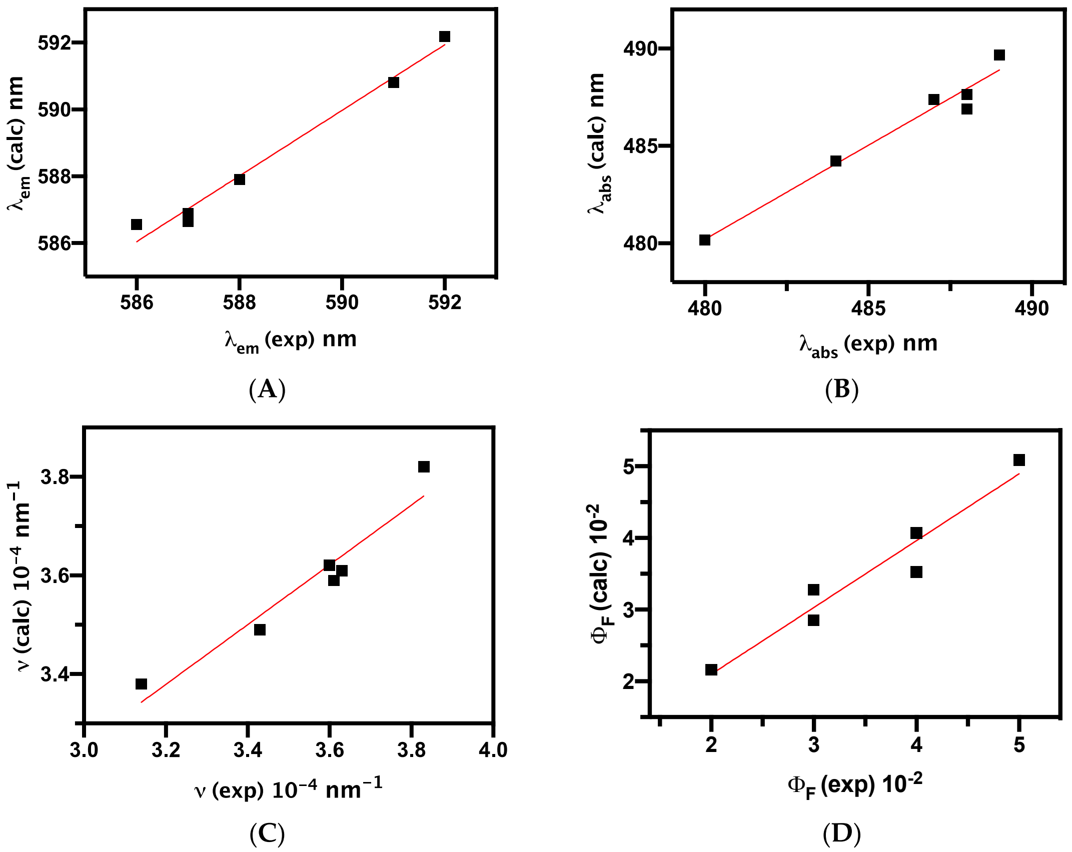

Figure S1. Linear relationship of the experimental and calculated (**A**) λ_{em} (R = 0.99); (**B**) λ_{abs} (R = 0.98); (**C**) $\Delta\tilde{\nu}$ (R = 0.97); and (**D**) Φ_F (R = 0.96) of Doxorubicin obtained by the multiple linear regression analysis according to Catalán solvent scale.

2. TEM image and DLS correlation curves of SMLs

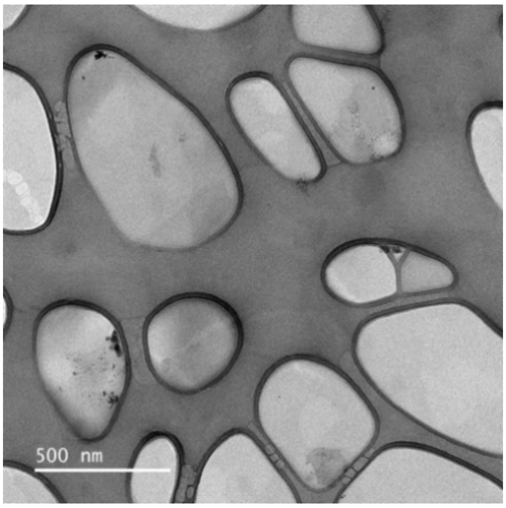

Figure S2. TEM image of solid magnetoliposomes at a lower magnification.

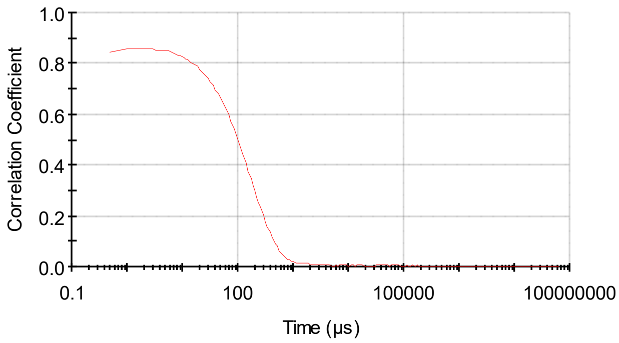

(A)

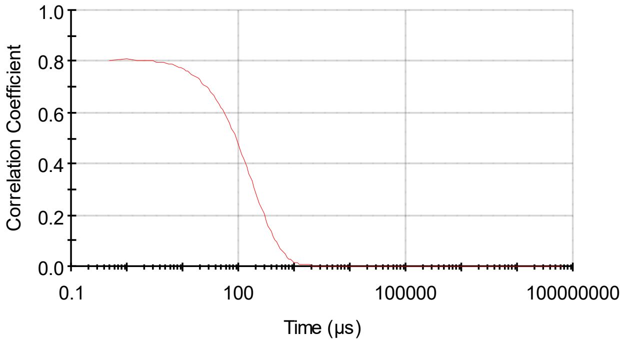

(B)

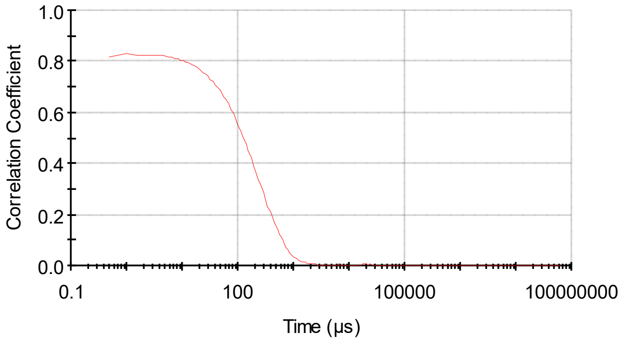

(C)

Figure S3. DLS correlation curves for solid magnetoliposomes. **A**: SMLs of DPPC; **B**: SMLs of DPPC/Ch; **C**: SMLs of DPPC/DSPE-PEG.

3. Drug release kinetics and mathematical modelling of release profile

The Weibull model, which is a distribution function, is expressed in terms of the drug fraction accumulated (m) in solution on the time t by Equation S.8 [11]:

$$m = 1 - exp^{-(t-T_i)^{\frac{b}{a}}} \quad (S.8)$$

where a is a scale parameter that defines the timescale of the process, T_i represents the latency time of the release process (often being zero), and b is a formal parameter that characterizes the type of curve ($b = 1$ is exponential; $b > 1$ is sigmoid, with ascendant curvature delimited by an inflection point; and $b < 1$ is parabolic, displaying high initial slope and a consistent exponential character).

The first-order kinetic model follows the Equation S.9 [12]:

$$F(\%) = M_0 \times (1 - e^{-kt}) \quad (S.9)$$

where F(%) is the percentage of released drug, M_0 represents the total amount of the drug released, k represents the first-order rate constant and t the time. Considering that the total drug release varied between experiments, M_0 was considered as a variable.

The Korsmeyer–Peppas model (power law) is a more comprehensive semi-empirical equation that establishes an exponential relationship between release and time, following Equation S.10 [13]:

$$ {}^{C_t}/_{C_0} = K \cdot t^n \qquad \text{(S.10)} $$

where C_0 and C_t are the concentrations at time 0 and t, respectively, K is the rate constant and n is the transport exponent. When $n < 0.45$, the release mechanism is diffusion-controlled (Fickian release), $0.45 < n < 0.89$ indicates a combination of diffusion and erosion drug release (non-Fickian release), $0.89 < n < 1$ indicates a relaxation-controlled release, and in the case of $n > 1$, the release is controlled by swelling and chain relaxation. The constant values and coefficient of determination obtained for each model are summarized in Table S3 (for DPPC-based SMLs) and Table S4 (for DPPC/DSPE-PEG-based SMLs).

Table S3. Obtained constant values by the fitting of each mathematical model to the kinetic data and respective coefficient of determination (R^2), according to the temperature and pH variation for DPPC-based SMLs.

	**Temperature**	y_{max}**(%) ± SD**	**Weibull**			**First-order**		**Korsmeyer-Peppas**		
			B	***a***	R^2	***k***	R^2	***K***	***n***	R^2
pH=5.5	42 °C	25 ± 2	1.18	0.29	0.99	0.35	0.99	10.76	0.30	0.88
	37 °C	9 ± 1	0.89	0.37	0.94	0.34	0.93	3.74	0.28	0.90
pH=7.4	42 °C	6.5 ± 0.2	1.59	0.71	0.96	0.82	0.94	4.66	0.14	0.74
	37 °C	4 ± 1	0.83	0.34	0.88	0.27	0.88	2.64	0.28	0.83

Table S4. Obtained constant values by the fitting of each mathematical model to the kinetic data and respective coefficient of determination (R^2), according to the temperature and pH variation for DPPC/DSPE-PEG-based SMLs.

	**Temperature**	y_{max}**(%) ± SD**	**Weibull**			**First-order**		**Korsmeyer-Peppas**		
			b	***a***	R^2	***k***	R^2	***K***	***n***	R^2
pH=5.5	42 °C	14 ± 1	0.14	0.93	0.99	0.34	0.99	2.570	0.52	0.98
	37 °C	5 ± 1	0.07	2.59	0.95	0.35	0.85	2.83	0.33	0.63
pH=7.4	42 °C	7 ± 3	0.34	0.88	0.87	0.31	0.87	1.87	0.33	0.93
	37 °C	9 ± 1	0.33	1.58	0.97	0.40	0.95	3.37	0.30	0.74

References

1. Inamdar, S.; Nadaf, Y.; Mulimani, B. Ground and excited state dipole moments of exalite 404 and exalite 417 UV laser dyes determined from solvatochromic shifts of absorption and fluorescence spectra. *J. Molec. Struct.* **2003**, *624*, 47–51.
2. Kawski, A.; Kukliński, B.; Bojarski, P. Excited state dipole moments of 4-(dimethylamino)-benzaldehyde. *Chem. Phys. Lett.* **2007**, *448*, 208–212.
3. Taft, R.W.; Abboud, J.-L. M.; Kamlet, M.J.; Abraham, M. H. Linear solvation energy relations. *J. Sol. Chem.* **1985**, *14*, 153–186.
4. Abboud, J.M.; Abraham, M.; Taft, R. Linear solvation energy relationships. 23. A comprehensive collection of the solvatochromic parameters, π*, α, and β, and some methods for simplifying the generalized solvatochromic equation. *J. Org. Chem.* **1983**, *48*, 2877–2887.
5. Catalán, J.; Díaz, C. Extending the Solvent Acidiy Scale to Highly Acidic Organic Solvents: The Unique Photophysical Behaviour of 3,6-Diethyltetrazine. *Eur. J. Org. Chem.* **1999**, *1999*, 885–891.

6. Marcus, Y. The properties of organic liquids that are relevant to their use as solvating solvents. *Chem. Soc. Rev.* **1993**, *22*, 409–416.
7. Catalán, J. Toward a generalized treatment of the solvent effect based on four empirical scales: Dipolarity (SdP, a new scale), polarizability (SP), acidity (SA), and basicity (SB) of the medium. *J. Phys. Chem. B* **2009**, *113*, 5951–5960.
8. Catalán, J.; López, V.; Pérez, P.; Martin-Villamil, R.; Rodríguez, J.-G. Progress towards a generalized solvent polarity scale: The solvatochromism of 2-(dimethylamino)-7-nitrofluorene and its homomorph 2-fluoro-7-nitrofluorene. *Liebigs Annal.* **1995**, *1995*, 241–252.
9. Catalán, J.; Díaz, C. A Generalized solvent acidity Scale: The Solvatochromism of o-*tert*-Butylstilbazolium Betaine Dye and Its Homomorph o,o′-Di-*tert*-butylstilbazolium Betaine Dye. *Liebigs Annal.* **1997**, *1997*, 1941–1949.
10. Katritzky, A.R.; Fara, D.C.; Yang, H.; Tämm, K.; Tamm, T.; Karelson, M. Quantitative measures of solvent polarity. *Chem. Rev.* **2004**, *104*, 175–198.
11. Noyes, A.A.; Whitney, W.R.; The rate of solution of solid substances in their own solutions, *J. Am. Chem. Soc*. **1987**, *19*, 930–934.
12. Papadopoulou, V.; Kosmidis, K.; Vlachou, M.; Macheras, P. On the use of the Weibull function for the discernment of drug release mechanisms, *Int. J. Pharm.* **2006**, *309*, 44–50.
13. Korsmeyer, R.W.; Gurny, R.; Doelker, E.; Buri, P.; Peppas, N.A. Mechanisms of solute release from porous hydrophilic polymers, *Int. J. Pharm.* **1983**, *15*, 25–35.
